# Site-Selectively Functionalized Albumin with DFO*Maleimide for ^89^Zr-Radiolabeling Yields a Metabolically Stable PET Probe that Enables Late Time-Point Tumor Imaging in Mice

**DOI:** 10.1021/acs.jmedchem.5c00803

**Published:** 2025-06-17

**Authors:** Julia Kronberger, Theresa Balber, Hemma Schueffl, Raphaela Wahrmann, Anja Federa, Mathias Gradl, Marie R. Brandt, Thomas Wanek, Markus Mitterhauser, Christian R. Kowol, Thomas L. Mindt, Petra Heffeter

**Affiliations:** † Institute of Inorganic Chemistry, Faculty of Chemistry, 27258 University of Vienna, Vienna 1090, Austria; ‡ Ludwig Boltzmann Institute Applied Diagnostics, General Hospital of Vienna, Währinger Gürtel 18-20, Vienna 1090, Austria; § Vienna Doctoral School in Chemistry, University of Vienna, Währinger Straße 42, Vienna 1090, Austria; ∥ Department of Biomedical Imaging and Image Guided Therapy, Division for Nuclear Medicine, 27271 Medical University of Vienna, Währinger Gürtel 18-20, Vienna 1090, Austria; ⊥ Center for Cancer Research and Comprehensive Cancer Center, Medical University of Vienna, Borschkegasse 8a, Vienna 1090, Austria; # Joint Applied Medicinal Radiochemistry Facility, University of Vienna, Medical University of Vienna, Vienna 1090, Austria; ∇ Department of Biomedical Imaging and Image Guided Therapy, Preclinical Imaging Laboratory (PIL), Medical University of Vienna, Vienna 1090 Austria

## Abstract

Human serum albumin (HSA) is a clinically validated drug carrier that improves drug delivery to tumor tissues. However, clinical imaging strategies are lacking to stratify patients who will benefit from HSA-bound drugs. In this study, we site-selectively radiolabeled HSA with zirconium-89 (^89^Zr), using the octadentate chelator DFO*, to provide an imaging probe with enhanced stability and sufficient half-life to elucidate the long-term (tumoral) albumin homeostasis. [^89^Zr]­Zr-DFO*malHSA demonstrated excellent metabolic stability and high tumor uptake in a longitudinal PET study (72 h p.i.) using a subcutaneous colorectal cancer allograft model (CT26). Preliminary results also showed enhanced enrichment of the PET probe in an intraperitoneally injected CT26 model indicating the role of the EPR effect not only in subcutaneous models. Consequently, [^89^Zr]­Zr-DFO*malHSA is a promising tool to image albumin accumulation in malignant tissues and should be further (pre)­clinically developed as a companion diagnostic agent for patient stratification in trials with albumin-binding drugs.

## Graphical abstract

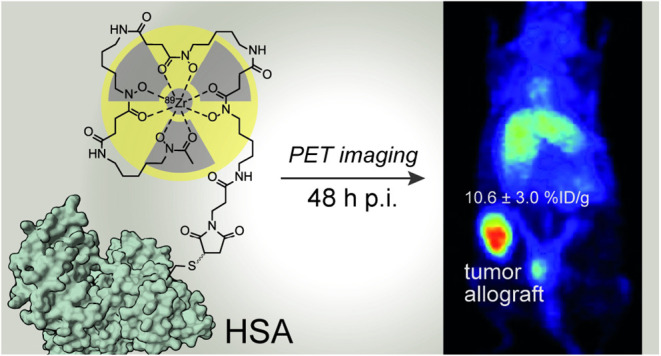

## 1 Introduction

Human serum albumin (HSA), as the most abundant plasma protein, constitutes about 60% of all serum proteins and is characterized by a remarkable blood plasma half-life in humans of around 19 days.^1,2^ This longevity can be attributed to constant cellular recycling via the chaperon neonatal Fc receptor (FcRn), which safeguards albumin (as well as IgG) against intracellular lysosomal degradation allowing exocytosis back to the bloodstream.^2,3^ Moreover, albumin is constantly reabsorbed from the glomerular filtrate by the proximal tubular cells of the kidneys.^4^ With diverse functionalities, albumin plays a pivotal role in regulating colloidal osmotic pressure, contributing to the antioxidant capacity of human plasma, and serving as a transporter for various molecules.^5,6^ Its role as a carrier protein stems from its multiple binding sites for e.g., hormones, fatty acids, amino acids, metal ions, bile acids, toxic metabolites (e.g., bilirubin), or drugs.^1,7^

Given its endogenous nature, albumin is nontoxic and nonimmunogenic, rendering it an appealing candidate as a drug carrier, enhancing blood circulation time and tumor accumulation of therapeutics and imaging agents.^8–12^ The increased uptake of albumin by tumors is predominantly attributed to distinct nutrient acquisition strategies employed by malignant cells, leading to heightened consumption and degradation of plasma proteins like albumin.^13^ Additionally, the enhanced permeability and retention (EPR) effect contributes to the increased accumulation of larger molecules (>40 kDa), including albumin, in malignant tissues.^14–16^ This effect results from the combination of enhanced vascular permeability with impaired lymphatic drainage and accumulation of phagocytic cells.^17^

The clinical success of albumin-based drug formulations underscores the potential of albumin for targeting tumors and metastases by enhanced drug delivery.^8,18–21^ Moreover, numerous preclinical studies have explored albumin-binding drugs, revealing promising potential applications.^8–10,21^ In more detail, there are currently two main strategies for the generation of albumin-targeting therapeutics. The clinically best-established strategy is based on the nanoparticle albumin-bound (nab) technology. The main representative of this strategy is Abraxane, which releases paclitaxel and has already been approved by the FDA against several cancer types. Currently, according to clinicaltrials.gov (search March 2025), about 350 clinical trials are ongoing with this therapeutic agent. Moreover, in 2021, Fyarro, an mTOR inhibitor-loaded albumin nanoparticle using the nab technology, was approved for the treatment of perivascular epithelioid cell tumors.^22^ On the other hand, several small-molecule albumin-targeting drugs have been developed. In particular, Aldoxorubicin has to be mentioned, which was successfully tested in a clinical phase 3 trial against soft tissue sarcoma (NCT02049905). Aldoxorubicin is a doxorubicin-releasing prodrug, which binds selectively to albumin via a maleimide moiety upon i.v. application.^23,24^ During the last years, we have focused our research on the development of albumin-targeted platinum prodrugs. Oxaliplatin-releasing prodrugs (e.g., KP2156 and KP2299) demonstrated exciting efficacy against colorectal cancer (CRC) in preclinical models, which prompted us to develop further this compound class toward first-in-man clinical trials.^25–28^

Noteworthy, although the development of albumin-based drug delivery concepts has been successful, promising albumin-targeting drugs like Aldoxorubicin failed clinical approval, so far.^20^ A main reason for this is the lack of suitable stratification tools to identify patients who would benefit from albumin-based therapy in a personalized manner. One powerful tool for preselecting cancer patients would be the assessment of the real-time tumoral albumin consumption rate by noninvasive nuclear imaging modalities such as positron emission tomography (PET) or single-photon emission computed tomography (SPECT). There has already been some exploration in this field:^11,29^ For example, HSA-derived PET tracers based on iodine-125/131 (^125^I and ^131^I),^30,31^ copper-64 (^64^Cu),^32,33^ gallium-68 (^68^Ga),^34–36^ zirconium-89 (^89^Zr),^37,38^ or SPECT tracers containing technectium-99m (^99m^Tc)^29^ or indium-111 (^111^In)^31,39^ have been investigated.

However, there are several reasons limiting the utility of those investigated albumin-based radiotracers: (1) The choice of the applied radionuclide: On one hand, SPECT has a lower resolution compared to PET and, on the other hand, the physical half-lives of some radionuclides are too short for late time point investigations.^34,35^ For example, clinically approved albumin-based nuclear medical imaging agents are up to now limited to ^99m^Tc-labeled human albumin macro-aggregates and nanocolloids used for lung perfusion imaging or sentinel lymph node mapping, respectively.^29^ However, there are already indications that albumin-mediated drug accumulation into tumor tissue takes place over several days.^27^ Consequently, due to the shorter physical half-life of ^99m^Tc (*t*_1/2_ = 6.02 h) or ^68^Ga (*t*_1/2_ = 68 min), such radiotracers are not suitable for imaging long-term tumor accumulation of albumin. Thus, a PET imaging tool, e.g., based on ^89^Zr (*t*_1/2_ = 3.3 d) is needed, which matches better the long plasma half-life of the protein. (2) The choice of the bioconjugation strategy: Site-selective functionalization of proteins is desired, as consequently the resulting bioconjugate should be only minimally altered compared to the native protein. However, some literature references report the nonsite-selective introduction of the chelator via abundant lysine residues for the labeling of albumin. This leads to a mixture of bioconjugates differing in the position and number of modifications and, consequently, impacts on the functionality of the protein.^37,38^ (3) The used chelator: In most cases, the known iron chelator DFO has been used for zirconium complexation.^37,40^ However, multiple reports indicate that the resulting complex [^89^Zr]­Zr-DFO lacks sufficient stability for long-term zirconium complexation over several days. When using DFO, slow release of the osteophilic [^89^Zr]­Zr^4+^ from the DFO complex is observed, limiting its applicability for long-term albumin imaging.^41,42^ The chemical reason is that DFO is a hexadentate chelator, while zirconium prefers a coordination number of eight. In order to allow the synthesis of ^89^Zr-based PET tracers with improved stability (most commonly for ^89^Zr-immunoPET)^43^, recently, a new chelator (DFO*) has been developed, which is superior to DFO for chelating zirconium. In more detail, in DFO*, the structure of DFO was extended by one hydroxamate unit,^44^ which leads to improved stability of the metal chelate *in vivo*.^42,45^ Consequently, DFO* is currently the only chelator commercially available for long-term ^89^Zr-PET imaging.^46^

Hence, the overarching objective of this study was to establish a novel PET imaging platform for patient stratification concerning albumin-binding drugs. In more detail, this study introduced a strategy for radiolabeling HSA with [^89^Zr]­Zr^4+^, utilizing the octadentate chelator DFO* as a superior chelating agent, to form radiometal complexes of higher *in vivo* stability, compared to the hexadentate chelator DFO, suitable for longitudinal tumor-imaging studies. HSA was site-specifically modified, using DFO*maleimide (DFO*mal) for conjugation to the free thiol of albumin at position 34 (Cys^34^). After ^89^Zr-radiolabeling, the radiotracer was analytically well-characterized and showed high stability in human blood serum *in vitro* and also in mouse serum *ex vivo*. Based on our expertise and interest in CRC, we aimed to perform *in vivo* validation using the murine CRC model CT26, which is well-characterized for its high albumin uptake and favorable response to albumin-bound prodrugs.^27^ CT26 was injected not only subcutaneously into mice but also in form of an orthotopic carcinosis model to demonstrate that the strategy is effective beyond subcutaneous tumors, which exhibit a pronounced EPR effect.

## 2 Results and Discussion

### 2.1 Characterization of Different Commercially Available HSA

First, various commercial sources of HSA were investigated as starting material for conjugation of DFO*mal and ^89^Zr-radiolabeling, focusing on the percentage of free thiol groups per albumin molecule, the presence of aggregates, and analysis of the uniformity of the protein. In order to obtain conjugates with a chelator-to-protein ratio close to one, a high amount of the reactive thiol group at Cys^34^ is important. In the healthy human body, 70–80% of HSA is present in its reduced form. However, processing and storage can lead to increased oxidation of Cys^34^ to sulfinic or sulfonic acids over time.^47^ 5,5′-Dithiobis­(2-nitrobenzoic acid) (DTNB) assays were performed to determine the fraction of free thiol of HSA (Table 1). Four different HSAs were investigated, a pharmaceutical formulation (HSA1) and three lyophilized powders (HSA2 and HSA3 from native human serum, and the recombinant HSA4). The pharmaceutical formulation HSA1 exhibited the highest proportion of free thiol groups per protein with approximately 41%.

**Table 1:** Available Free Thiol Groups Per Protein Molecule as Determined by DTNB Assays^a^

HSA	origin	formulation	% free thiol
1	native	solution	41.4 ± 0.8
2	native	lyophilized	23.2 ± 0.6
3	native	lyophilized	36.9 ± 0.4
4	recombinant	lyophilized	26.7 ± 1.2

a Experiments were performed in triplicates (*n* = 2)

In addition, size-exclusion chromatography (SEC) was conducted to assess the protein purity and presence of aggregates via absorbance at λ = 280 nm. Chromatograms can be found in the Supporting Information (Figures S1–S4). Apart from the albumin monomer, the dimer and the trimer^48^ could also be detected to some extent (Table 2). Notably, in preparations HSA2 and HSA4 the degree of dimerization was over 10%, making them unfavorable for functionalization compared to HSA1 and HSA3.

**Table 2:** Aggregate Contents of Different Commercially Available HSA According to SEC UV Trace at λ = 280 nm^a^

HSA	HSA monomer (rt)	HSA dimer (rt)	HSA trimer (rt)
1	96.0% (8.1 min)	1.1% (7.1 min)	2.9% (5.6 min)
2	89.3% (7.6 min)	10.7% (6.7 min)	n.d.
3	96.7% (7.9 min)	3.4% (7.3 min)	n.d.
4	89.8% (8.1 min)	10.2% (7.2 min)	n.d.

a n.d. = not detected, rt = retention time.

HSA can exist in various adduct forms, resulting from covalent, noncovalent, and coordinative binding to different molecules and metals, depending on its origin, processing, and handling. To assess the uniformity of the investigated HSA, electrospray ionization mass spectrometry (ESI-MS) analysis was performed and the width of the range of signals was used as an indicator for the homogeneity of the protein. The deconvoluted mass spectrum of HSA3 showed a signal range of about 0.5 kDa, HSA1 of about 0.6 kDa, HSA2 of about 1.8 kDa, and HSA4 of over 2.0 kDa. Mass spectra can be found in the Supporting Information (Figures S5–S8). Consequently, we did not consider the less homogeneous HSA2 and HSA4 for our studies.

The high availability of reduced Cys^34^, the low content of aggregates according to SEC, and the sufficient homogeneity as determined by mass spectrometry (MS) analysis, prompted us to choose the pharmaceutical albumin formulation HSA1 for the conjugation of DFO*mal and subsequent ^89^Zr-radiolabeling.

### 2.2 Synthesis, Radiolabeling, Quality Control, and Stability of [^89^Zr]­Zr-DFO*malHSA

In the living body, HSA is permanently controlled for modifications.^49^ When the protein structure is altered to a certain extent, this leads, on the one hand, to increased recognition by degradation-inducing receptors such as gp18 and gp30. On the other hand, binding of HSA to the intracellular chaperon FcRn can be hampered leading to lysosomal degradation.^19^ However, as demonstrated by the clinical success of albumin-binding drugs, several sites on HSA are known to tolerate the binding of small molecules. One of these is the Cys^34^, which probably can be explained by the fact that this binding site is also important for the physiological transport of endogenous molecules.^1^ Accordingly, this binding site has been successfully used for drug conjugation via a maleimide moiety in case of Aldoxorubicin,^50^ KP2156,^25,27^ and KP2299.^28^ Based on available data in the literature, indicating a high stability of these albumin-drug conjugates *in vivo*,^27,50^ we also choose a maleimide moiety as the albumin-binding unit for the new radiotracer. This moiety allows fast and selectively binding to the thiol of Cys^34^ of albumin under physiological conditions.^51^ Consequently, even though the *in vivo* stability of the maleimide–thiol linkage has been controversially discussed in the literature, it has found successful clinical application in several approved antibody drug conjugates (ADCs), such as Brentuximab vedotin (Adcetris) or Trastuzumab emtansine.^52^

The bioconjugate DFO*malHSA was synthesized using modified published procedures.^53^ DFO*mal was dissolved in dimethyl sulfoxide (DMSO) at 37 °C. Subsequently, HSA in phosphate-buffered saline (PBS, pH 7.4, SEC chromatogram shown in Figure S9) was added to achieve <5% w/w DMSO (Scheme 1).^53^ The mixture was gently shaken at 37 °C for 45 min. After purification and reformulation to 0.9% NaCl via PD-10 column and spin-filtration, the product was successfully obtained according to SEC and ESI-MS data. SEC confirmed the protein purity of the albumin monomer and monomer conjugate >95% at λ = 280 nm after purification, whereas the other 5% of the UV trace corresponds to the albumin dimer and trimer (Figure S10). The relative amount of dimer and trimer was not increased in comparison to the starting material. Hence, our modification did not result in further aggregation. Due to their similar size, it was not possible to distinguish HSA from the conjugated DFO*malHSA by SEC. With this method, we could only verify the integrity of the protein in terms of size, possible formation of aggregates, and disintegration. ESI-MS was conducted to show the successful bioconjugation for DFO*malHSA. MS revealed the main mass peak of the unconjugated albumin at approximately 66 437 Da, and a second peak at approximately 67 350 Da, the mass difference of which corresponded to the mass of the chelator DFO*mal (912 Da, Figure S11).^48^ This confirmed that a maximum of one chelator per protein molecule was present.

**Scheme 1:**
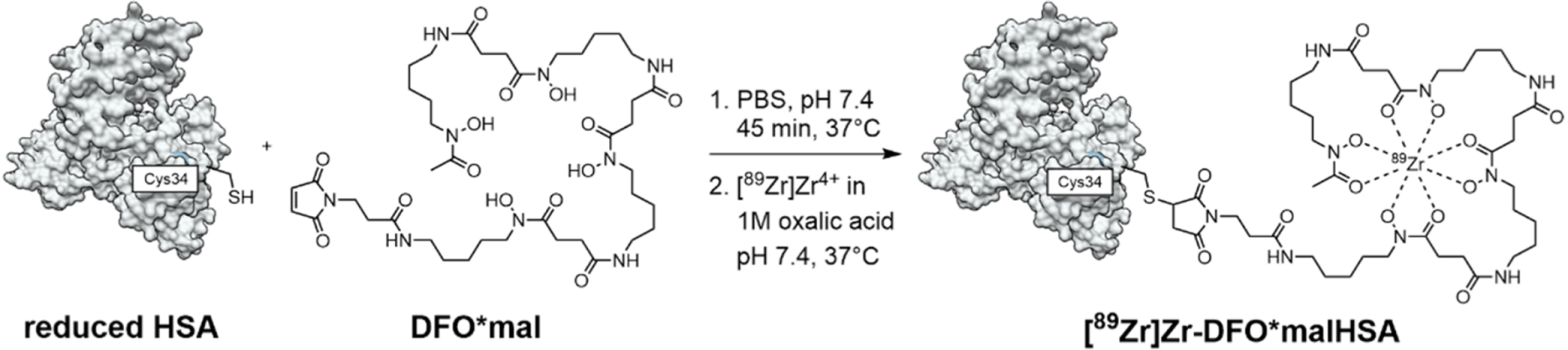
Bioconjugation of HSA and DFO*mal and Radiolabeling with [^89^Zr]­Zr^4+^ ^a^

a The structure of HSA was rendered from PDB 1AO6 ^54^ using Mol*^55^ (size of HSA and DFO*mal are not depicted proportionally).

Radiolabeling of DFO*malHSA with [^89^Zr]­Zr-oxalate was found to be facile and reproducible at 37 °C in 0.5 M HEPES buffer at pH 7.4 (Scheme 1), conditions which were previously reported for the radiolabeling of DFO*-modified antibodies.^42^ The crude radiolabeling solution was purified and thereby reformulated in 0.9% NaCl in two steps: First, via PD-10 SEC and second, with preparative spin filtration. The quality control of [^89^Zr]­Zr-DFO*malHSA comprised three methods: instant thin-layer chromatography (iTLC), SEC, and spin filter analysis. With iTLC, the free radionuclide fraction was quantified (*r*_f_ = 1), but not the fraction of radiolabeled chelator (*r*_f_ = 0), which could potentially be released from the protein ([^89^Zr]­Zr-DFO*malHSA *r*_f_ = 0, Figure S12). There are no reports on iTLC systems which allow radiolabeled DFO* to migrate with the solvent on a TLC plate. Hence, degradation products including DFO* could not be distinguished from intact radiotracer via iTLC. SEC provided qualitative information about successful radiolabeling of the protein, by having coincidental signals in the UV chromatogram and the radio channel (Figures S13–S17). However, we observed that released radiolabeled chelator (as well as free zirconium) did not elute quantitatively from the SEC column, probably due to precipitation of low-molecular weight species. A reliable determination of the radiochemical purity (RCP) was only possible via spin filtration analysis in addition to iTLC and SEC as previously reported.^42^ The molecular weight cutoff of the used spin filters (30 kDa) enables the separation of low-molecular weight species from the protein. Unspecific binding of [^89^Zr]­Zr^4+^ and radiolabeled DFO*mal to the filter membrane was <1%.

The crude radiolabeling solution showed a RCP and a crude radiochemical yield (RCY) of 68 ± 17% and 13 ± 14% of free [^89^Zr]­Zr^4+^. Using a PD-10 column in the first purification step, a RCP of 81 ± 9% was achieved, and the amount of free [^89^Zr]­Zr^4+^ reduced to 3 ± 2%. To reach >99% RCP and <1% free [^89^Zr]­Zr^4+^, three circles of spin-filter centrifugation were required as second purification step.

The final, decay-corrected RCY of [^89^Zr]­Zr-DFO*malHSA was 61 ± 19% (*n* = 16) with a RCP of >99%. The apparent specific activity of the radiolabeled product depended on the used amount of [^89^Zr]­Zr-oxalate and ranged from 5.6–26.9 MBq mg^–1^ for lower amounts of radioactivity (0.5–20 MBq). Up to 163.5 MBq mg^–1^ were achieved with higher amounts of radioactivity (70–160 MBq).

Next, long-term and short-term *in vitro* stability tests of the radiotracer [^89^Zr]­Zr-DFO*malHSA were performed (Figures 1 and 2). The time span of up to 7 d was applied for obtaining an estimation of the *in vivo* stability (in human serum) and in the formulation buffer (0.9% NaCl) for an assessment of the shelf life of the radiolabeled product. The shorter time span of up to 4 h was examined to ascertain stability during cell experiments. The stability was quantified by spin filter analysis as well as iTLC. Release of the radionuclide was never observed by iTLC (data not shown). Instability could only be seen via the formation of radioactive fragments smaller than 30,000 Da, which were detected by spin-filter analysis. We assume that these fragments were the radiolabeled chelator cleaved from the protein, presumably as the result from the known retro Michael reaction.^56^ Over 7 days, [^89^Zr]­Zr-DFO*malHSA remained >95% intact in 0.9% NaCl and >85% in human blood serum (Figure 1). Over 4 h, the use of RPMI cell culture medium led to the release of the chelator from the protein, and after 4 h, only 70% remained intact. Without 5% CO_2_ atmosphere, RPMI cell culture medium has a basic pH of 8.5 and is composed of a variety of substances with functional groups that could interfere with the succinimidyl thioether linkage, both of which could influence the stability of the radiotracer. Correlation between the pH of the medium and the radiotracer’s stability is assumed, as the tracer was stable in neutral to slightly acidic media. The cell culture medium contains an array of diverse free amino acids at concentrations reaching up to the millimolar range, which may compete with the radiotracer present at nanomolar concentrations. In 0.9% NaCl, human blood serum and in Hanks’ balanced salt solution containing 7 mM 2-morpholinoethanesulfonic acid (HBSS + MES buffer), the RCP remained >99% over 4 h (Figure 2).

**Figure 1:**
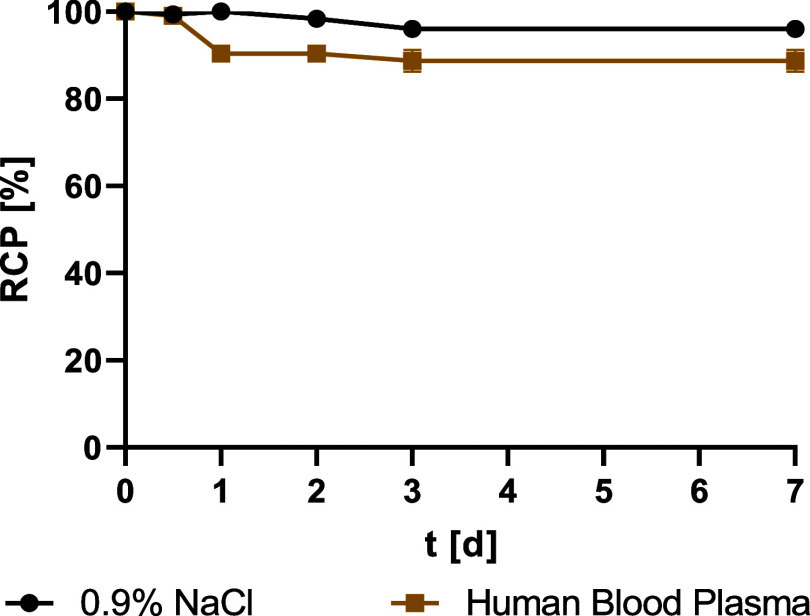
Stability (RCP) of [^89^Zr]­Zr-DFO*malHSA over 7 days in 0.9% NaCl and human blood plasma (*n* = 3 per time point, error bars are too small to be visualized).

**Figure 2:**
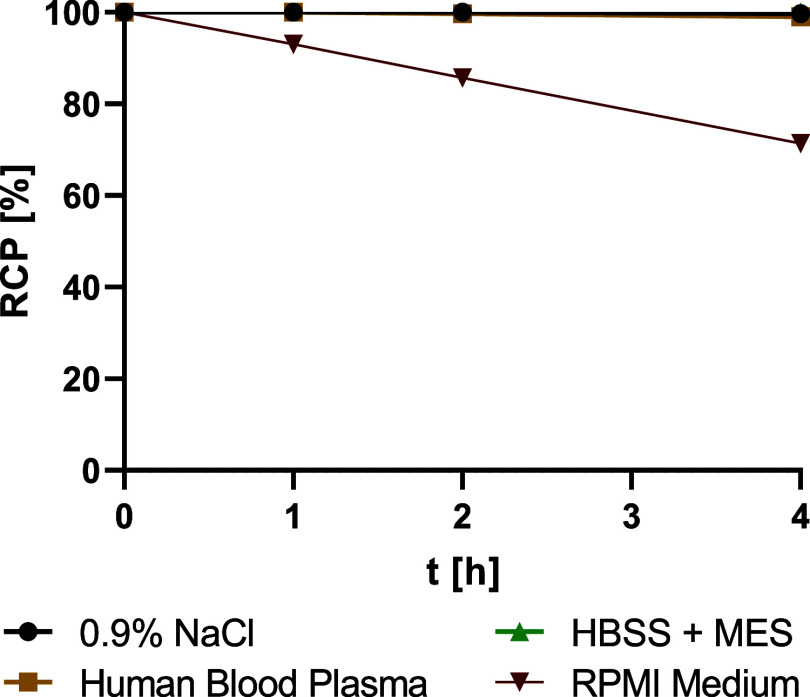
Stability of [^89^Zr]­Zr-DFO*malHSA over 4 h in 0.9% NaCl, human blood plasma, HBSS + MES buffer and RPMI-1640 cell culture medium (*n* = 3 per time point, error bars are too small to be visualized). Lines corresponding to stabilities in NaCl, blood plasma and HBSS+MES medium are overlapping.

### 2.3 Cellular Uptake of Fluorescent or Radiolabeled HSA

Based on already available expertise with the cell models^27^ and our future plans to apply the novel albumin-based radiotracer for the stratification of CRC patients for treatment with albumin-binding oxaliplatin derivatives (e.g., KP2156 and KP2299), we chose CRC as tumor model for the first biological investigations of [^89^Zr]­Zr-DFO*malHSA. Due to the instability of [^89^Zr]­Zr-DFO*malHSA in RPMI medium, all *in vitro* experiments were conducted in HBSS + MES buffer, in which the radiotracer was stable (Figure 2). In a first step, the influence of the HBSS + MES buffer on the albumin uptake of the human CRC model SW480 was evaluated using FITC-labeled HSA. To confirm that the increase of fluorescence was indeed via active transport of the labeled albumin (and not by, e.g., passive diffusion of liberated FITC), the experiment was performed at 4 °C in parallel. The results demonstrate an active and robust uptake of FITC-labeled HSA in HBSS+MES buffer (Figure S18), with ∼ 95% of cells showing FITC-associated fluorescence at 37 °C. This effect could be significantly reduced by incubating at 4 °C, further confirming its dependence on an active transport mechanism.

As a next step, SW480 cells were evaluated for their uptake of [^89^Zr]­Zr-DFO*malHSA. Noteworthy, for the detection of radiotracers, different experimental setups are necessary. For flow cytometry, a semiquantitative approach, the relative fluorescence of the treated samples is compared with the autofluorescence of untreated cells in suspension in a single-cell manner. When investigating radiolabeled compounds, the gold standard is to quantitatively compare internalized versus remaining free fractions in relation to each other, which allows (in contrast to flow cytometry) absolute quantification of the uptake of radiolabeled HSA conjugates. Disappointingly, in these settings, only low and inconsistent cell-associated albumin uptake (about 1%) was observed (Figure S19).

Although these results are contradictory to the ones obtained with FITC-labeled HSA, they are in line with previous reports on [^89^Zr]­Zr-DFO-HSA, where no significant amounts of radioactivity were detected in or bound to TZM-bl or PC-3 cells *in vitro*.^38^ The two fundamentally different experimental settings can probably explain this discrepancy between the cellular uptake of fluorescently labeled and radiolabeled HSA. While the setup with radiotracers allows the generation of quantitative results, it also leads to a higher limit of detection in the cell-associated fraction. Furthermore, using HBSS+MES as the buffer system restricts the incubation time to just a few hours, as prolonged exposure to this medium is not well tolerated by the cells. For longer incubation times like 24 h, a cell culture medium like RPMI would be necessary, which could not be used due to the instability of [^89^Zr]­Zr-DFO*malHSA as described above. Consequently, we concluded that no further information could be derived from cell experiments, which prompted us to continue with the evaluation of the new radioactive conjugate *in vivo* using tumor-bearing mice.

### 2.4 *In Vivo* PET/CT Imaging of a Subcutaneous Tumor Model

PET/CT imaging was performed in seven CT26-bearing Balb/c mice that received 8.02 ± 2.9 MBq [^89^Zr]­Zr-DFO*malHSA (corresponding to 26–78 μg protein) via tail vein injection. We selected this allograft model for experiments to evaluate our new radiotracer in an immunocompetent system. CT26 allografts are well-established in our laboratory, and data on pharmacokinetics and biodistribution of albumin-binding drugs, such as KP2156, are available for comparison.^27^ Figure S20 demonstrates that the cellular albumin uptake in both colorectal tumor cells CT26 (murine) and SW480 (human) is indeed similar.

Animals were either sacrificed after imaging (and organs were measured in a γ-counter) or reimaged at later time points. Four out of seven animals were repeatedly measured using PET/CT yielding 11 data sets in total. Representative images for each time point are shown in Figure 3 and the complete animal data is presented in Figures S21–S23. PET images acquired 30 min after radiotracer injection (*n* = 2) primarily showed the blood pool, e.g., uptake in the heart (21.4 ± 1.4%ID/cc), aorta, and carotids, which is in line with previously published data (Figure 3A).^38^ After 24 h, radioactivity in blood circulation was significantly reduced and started to accumulate in CT26 tumors (7.9 ± 2.6%ID/cc, *n* = 3, Figure 3B). Subsequently, [^89^Zr]­Zr-DFO*malHSA was retained in the tumor tissue for up to 72 h (7.3 ± 1.7%ID/cc at 48 h and 6.4 ± 1.4%ID/cc at 72 h, *n* = 3 each) (Figure 3C,D) and image contrast (tumor/background ratio) improved over time. Liver uptake could be observed starting from 24 h p.i. (6.7 ± 2.1, *n* = 3), indicating physiological albumin degradation, but was less prominent at this time point than expected from literature data.^37^ Noteworthy, radioactivity detected in the joints (knee and elbow) was <2%ID/cc at 72 h (see Table S1) indicating negligible amounts of free osteophilic [^89^Zr]­Zr^4+^,^42,46,57–59^ confirming high stability of the ^89^Zr-complex *in vivo*. Overall, the novel [^89^Zr]­Zr-DFO*malHSA yielded high image contrast for up to 72 h, the latest imaging time point ever reported for a radiolabeled albumin conjugate and indicated very promising tumor-targeting properties.

**Figure 3:**
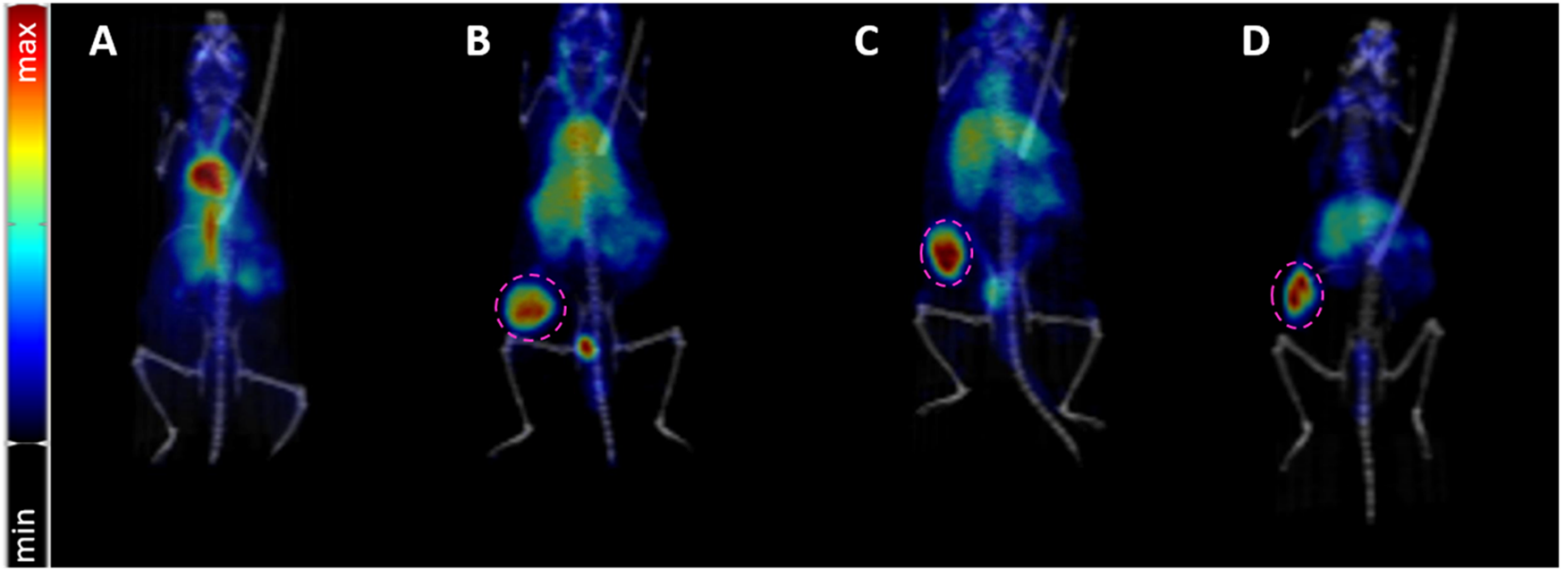
Representative maximal intensity projection (MIP) PET images fused with CT of Balb/c mice bearing subcutaneous CT26 tumors are shown for 30 min p.i. (A), 24 h p.i. (B), 48 h p.i. (C) and 72 h p.i. (D). PET data is decay corrected for the start of acquisition.

### 2.5 *Ex Vivo* Biodistribution, Plasma Stability and Cellular Uptake

To increase the number of animals investigated, a second experiment was performed for quantitative assessment of the biodistribution using *ex vivo* γ-counting of collected organs. As this is a very sensitive method, a much lower dose (compared to PET/CT imaging) could be administered. The biodistribution data for all animals of both experiments is summarized in Table S2. In general, no differences in biodistribution (%ID g^–1^ tissue) between animals that received low doses (0.27–1.6 MBq and 2.2–10.5 μg) and those that were subjected to PET/CT (3.1–12.4 MBq and 26–78 μg) were observed (Tables S3 and S4). This indicates that the tumoral albumin accumulation is based on a nonsaturable, passive drug-targeting mechanism, e.g., the EPR effect.^60^ Consequently, this allowed us to pool the data collected from both experiments (Table S2). As shown in Figure 4, biodistribution determined by the organ collection followed the same pattern as observed by *in vivo* PET imaging (compare Figures 3, S22 and S23). In more detail, after 30 min p.i., the highest radioactivity was found in the blood and in highly perfused organs such as the heart, lung, liver, kidneys, and spleen (blood pool). There was no significant tumor accumulation of [^89^Zr]­Zr-DFO*malHSA after 30 min. Within 24 h p.i., the radiotracer was significantly cleared from the blood and tumor uptake increased to 9.1 ± 1.8%ID g^–1^. Tumor uptake remained unchanged until the latest measured time point (10.6 ± 3.0%ID g^–1^ at 48 h p.i. and 7.6 ± 1.6%ID g^–1^ at 72 h p.i.), while we observed a slight but significant (*p* < 0.05) increase in liver uptake at the later time points. As a result, tumor/blood ratios are steadily increasing over time, while tumor/liver and tumor/kidney ratios remain approximately constant at 24, 48, and 72 h (Figure 5). It is well-known that the albumin concentration in the brain is very low compared to other tissues and blood.^61^ Consequently, the brain served as a negative control in this study. Indeed, only minimal signals were detected after 30 min, which further dropped at later time points. Noteworthy, there was also a very low uptake of the new radiotracer into the colon tissue, which is important for its future use for imaging of CRC patients. The biodistribution experiments showed a negligible ^89^Zr-upake in the femur at all examined time points (confirming the findings of PET imaging) confirming published data that [^89^Zr]­Zr^–^DFO* is highly stable *in vivo*. This finding is especially important, as it indicates stable complexation of [^89^Zr]­Zr^4+^ by the chelator DFO* up to 72 h.

**Figure 4:**
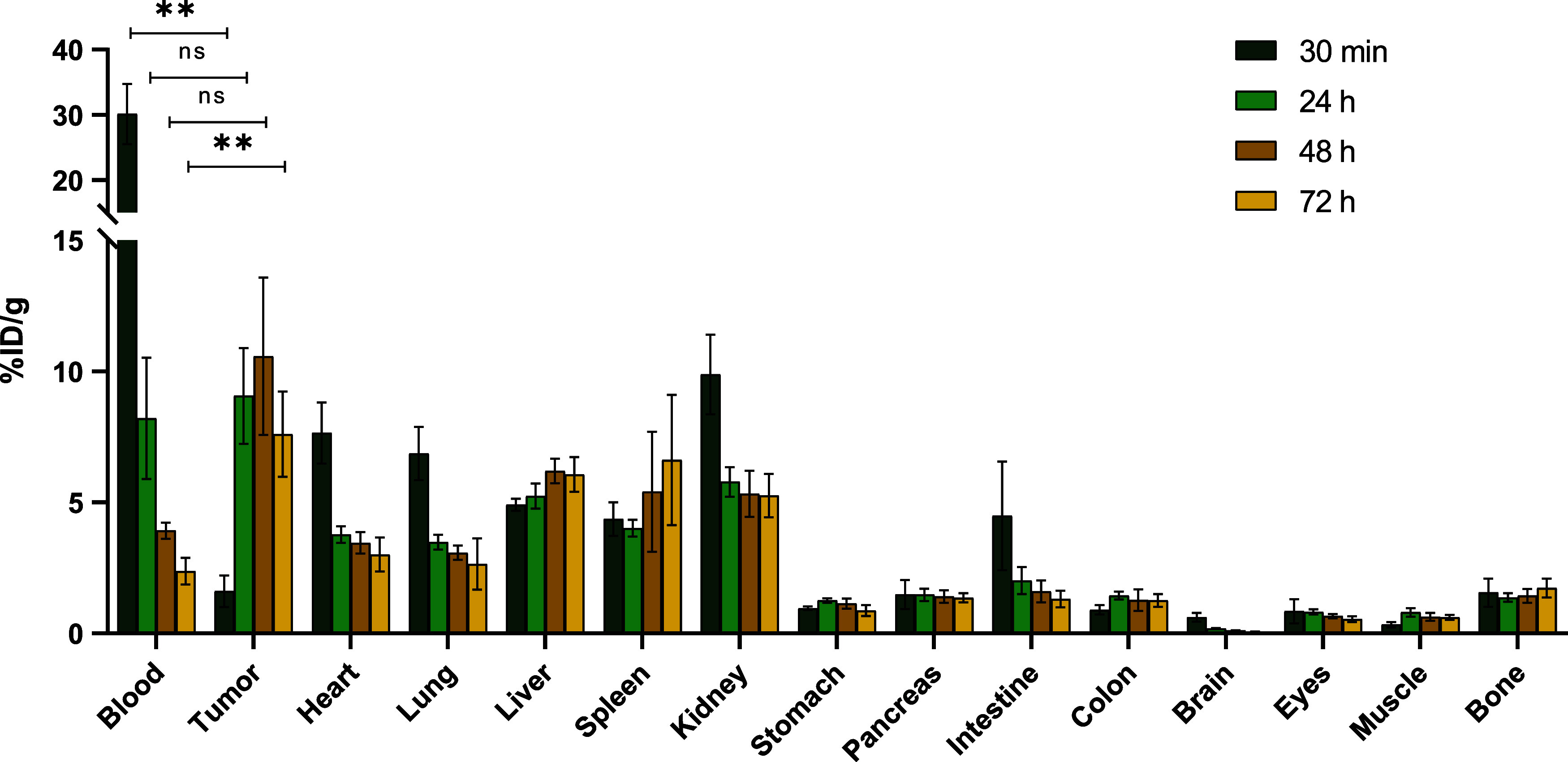
Distribution of [^89^Zr]­Zr-DFO*malHSA is depicted as %ID g^–1^ tissue for different organs and time points (*n* = 4 for 30 min and 24 h p.i., *n* = 5 for 48 h and *n* = 6 for 72 h p.i.). Decay correction was performed for the time of injection. Statistical significance between the time points of blood and tumor was tested by two-way ANOVA and multiple-comparison analysis (** = *p* < 0.01, ns = not significant).

**Figure 5:**
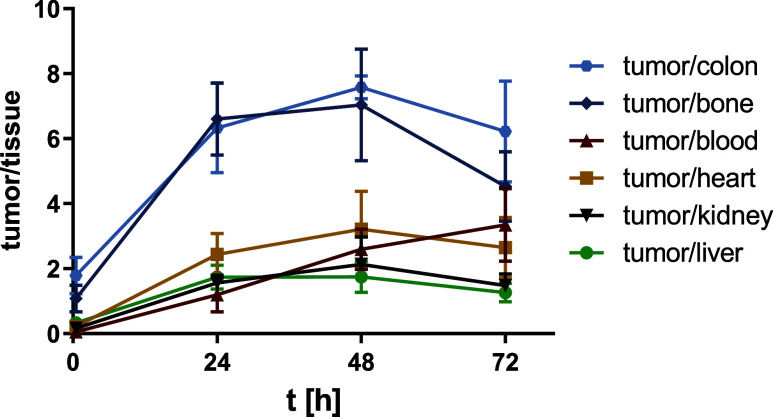
Tumor/tissue ratios of [^89^Zr]­Zr-DFO*malHSA over time.

Biodistribution data of [^89^Zr]­Zr-DFO* was investigated by Chomet et al. before in healthy mice.^45^ According to this study, the radiometal complex is cleared renally within 1 h p.i. and therefore does not interfere with our late-time point imaging study. To investigate further the *in vivo* serum stability of [^89^Zr]­Zr-DFO*malHSA, the collected serum samples of the mice were processed using spin-filter analysis as described above. These measurements revealed that up to the latest tested time point of 72 h, ≥99% of the radiotracer was intact and [^89^Zr]­Zr^4+^ was bound to albumin.

In addition to the γ-counting measurements of the tumor tissue, the cellular internalized [^89^Zr]­Zr-DFO*malHSA was determined after generating a single-cell suspension using a tumor digestion kit. For this experiment, four tumors were used (two of the 48 h and two of the 72 h cohort from the high-dose experiment). The results showed that about 10 ± 1.4% of the radioactivity was detected inside the tumor cells. There was no difference in the cellular uptake between 48 and 72 h. These preliminary data are interesting, as they could indicate that the enrichment of HSA in the extracellular space of the tumor due to the EPR effect results in a long-term depot in the tissue.^62^

As mentioned in the introduction, there are only a few reports in the literature that can be directly compared to our study. A number of studies focus on short-lived radionuclides^34–36^ or use albumin from other species (e.g., bovine serum albumin).^32^ To our knowledge, there are only two studies, which investigated ^89^Zr-labeled albumin, however, using DFO as a chelating moiety and performing unspecific bioconjugation of the chelator via lysine residues.^37,38^ In good agreement with our data, mainly blood pool imaging was observed within 24 h. However, in both studies the biodistribution imaging was stopped after maximum 24 h, making it impossible to compare the stability and tumor uptake of the radiotracer after several days. There are only two studies which investigate albumin imaging in mice for more than 24 h; (1) Park et al, who prepared a ^64^Cu-labeled HSA/folic acid conjugate (^64^Cu *t*_1/2_ = 12.7 h) using a lysine-based linker.^33^ The biodistribution of this radiotracer is rather different from our [^89^Zr]­Zr-DFO*malHSA. This observation could be attributed to the additional folic acid moieties, which may alter albumin in a way that prevents its binding to FcRn resulting in its lysosomal degradation. Moreover, the chelator was attached unspecifically to HSA via lysine moieties, which leads to an inhomogeneous mixture of bioconjugates.^63^ (2) The study by Daum et al, which reports an ^111^In-based HSA radiotracer via a maleimide linker for SPECT imaging.^39^ In this study, DTPA was used as a chelating moiety. Although the authors report some tumor-targeting of the radiotracer, the tumor levels never exceeded those of, e.g., the kidneys, which is in sharp contrast to the distinct tumor selectivity, high uptake and excellent tumor-to-background ratio of our [^89^Zr]­Zr-DFO*malHSA. Both studies reported a constant time-dependent decrease of radioactivity in the liver after 48 h, which appears to contradict our data. This prompted us to investigate the metabolic fate of unlabeled HSA in our mouse model in more detail.

### 2.6 Histological Evaluation of HSA Biodistribution in CT26-Bearing Balb/c Mice

To understand better the long-term stability and metabolic fate of [^89^Zr]­Zr-DFO*malHSA, we assessed the general biodistribution of unlabeled HSA in our CT26 mouse model. To this end, an antibody specific for HSA was exploited, which had no cross-reactivity with the endogenous murine albumin (Figure S24). The intensity of the stain could then be quantified over the whole slide using HALO software. In more detail, 12 CT26-bearing mice were injected with the commercially available HSA1 and the tissues collected after 30 min, 24 h, 48 h and 72 h. After formalin fixation and paraffin embedding, slices of 1.5 μm thickness were prepared and immunohistologically stained. As shown in Figures 6 and S25, the HSA pattern widely aligned with the one of [^89^Zr]­Zr-DFO*malHSA, proving the accumulation of intact HSA in malignant tumors, organs and other tissues. Interestingly and in contrast to the radiotracer, the HSA levels in the liver decreased over time. This effect correlated with the appearance of individual cell islands with a strong positive intracellular HSA signal (Figure S26). Together with the observation of a positive ^89^Zr-signal in the bladder of several [^89^Zr]­Zr-DFO*malHSA-treated animals, we hypothesize that our data indicate a normal physiological degradation of the applied HSA and [^89^Zr]­Zr-DFO*malHSA, respectively, in the hepatocytes (which has a half-life of ∼ 1.5–2 days in mice) with renal excretion of (radioactive) metabolites as a consequence.^64^ As relevant radioactivity levels were at no time point detected in the bones of the treated animals, we assume that even after degradation of HSA in the liver, the [^89^Zr]­Zr^4+^ remains complexed by DFO* and does not get released.

**Figure 6:**
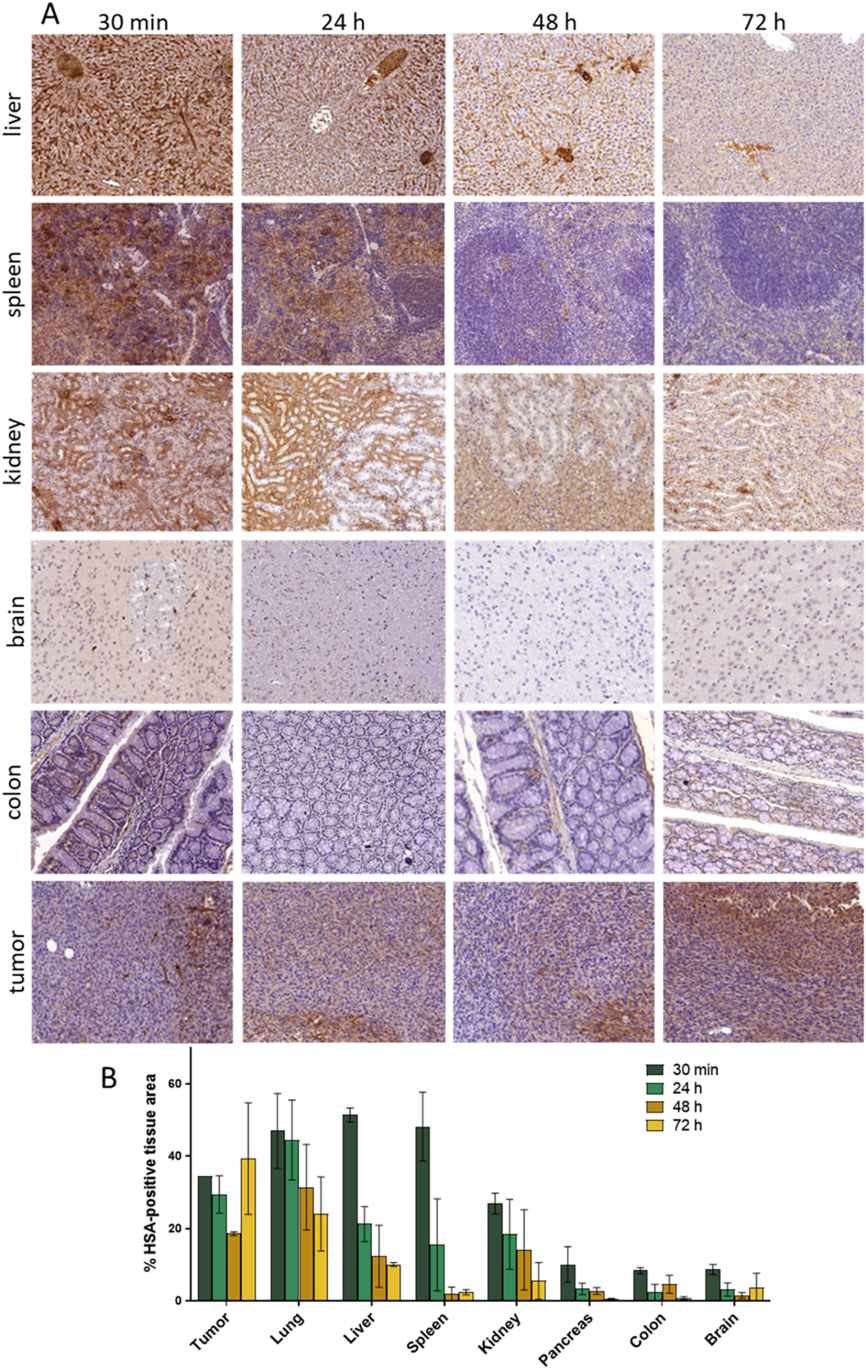
Histological evaluation of HSA distribution in Balb/C mice bearing CT26 tumors. Tumor-bearing animals were treated with HSA (1 g/kg) i.v. After 30 min, 24 h, 48 h and 72 h (*n* = 3 per time point) tumors and different organs were harvested and further processed for HSA-specific immunohistological staining. (A) representative histological scan. (B) Quantification of % HSA-positive area analyzed by HALO software. Values given are means ± SD.

### 2.7 *In Vivo* PET/CT Imaging of a Carcinosis Model

After successful demonstration of enhanced accumulation of [^89^Zr]­Zr-DFO*malHSA in subcutaneous tumor allografts, the next objective was to investigate whether this effect also occurs in a more physiological model. To this end, a pilot study was conducted, wherein F-luciferase-transfected CT26 cells were intraperitoneally injected into a female Balb/c mouse (*n* = 1) to mimic carcinosis, which frequently occurs at the late stage of the disease. 8.07 MBq [^89^Zr]­Zr-DFO*malHSA were applied and the PET images obtained 48 h p.i. revealed enhanced accumulation of [^89^Zr]­Zr-DFO*malHSA in the tumor tissue (Figure 7A). Tumor localization was confirmed post-mortem by bioluminescence imaging (Figure 7B). Tumor nodules and organs were collected and quantified using γ-counting (Figure S27 and Table S5), indicating a radiotracer accumulation of 7.1 ± 0.6% ID g^–1^ in the tumor nodules in the same range as the s.c. tumors in the first experiment. Noteworthy, the tumoral uptake of [^89^Zr]­Zr-DFO*malHSA was very homogeneous over the four collected tumor nodules, indicating that albumin uptake is not impacted by the individual localization and size of the tumor. This preliminary finding underscores the potential of the new ^89^Zr-labeled HSA, even in orthotopic tumor models. Nonetheless, further experiments employing PET/MRI are necessary to delineate better tumor tissue from healthy tissue. To the best of our knowledge, this is the first report on albumin PET imaging in an intraperitoneally injected tumor model.

**Figure 7:**
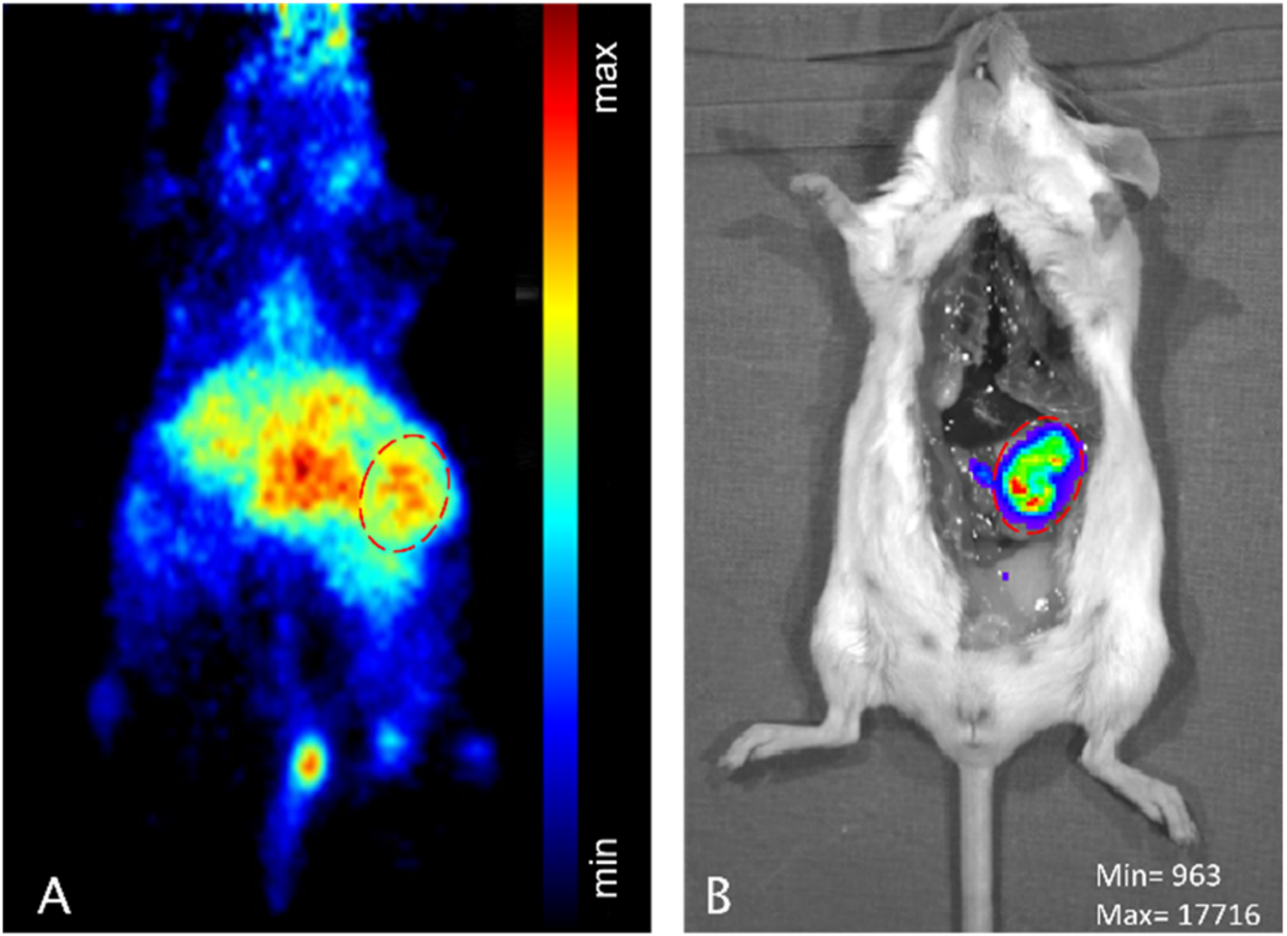
F-luciferase-transfected CT26 cells were intraperitoneally injected into a female Balb/c mouse. After 8 days of tumor growth, 8.07 MBq [^89^Zr]­Zr-DFO*malHSA was i.v. injected and PET imaging was performed 48 h p.i. Maximal intensity projection (MIP) is shown (A). Bioluminescence imaging was performed post-mortem as anatomical reference (B).

## 3 Conclusions

Despite the successful clinical establishment of albumin-binding anticancer drugs like Abraxane, understanding the role of albumin in cancer homeostasis and identifying biomarkers for targeted drug delivery remains challenging. Reported examples of albumin-based imaging approaches for potential patient stratification have been only of limited success and thus novel tools like PET radiotracers for better assessment and utilization of albumin-targeting drugs are needed. In this study, we successfully synthesized a well-defined and characterized ^89^Zr-labeled albumin imaging agent with excellent *in vitro* and *in vivo* stability, enabling longitudinal PET imaging of subcutaneous tumor allografts in mice for up to 72 h. The increased tumor uptake and retention of [^89^Zr]­Zr-DFO*malHSA at late time points has not been reported yet and is consistent with the suggested molecular mechanisms underlying tumoral albumin homeostasis, likely enabled by the EPR effect. Moreover, preliminary results showed successful PET imaging of [^89^Zr]­Zr-DFO*malHSA also in an intraperitoneally injected tumor indicating the enhanced tumoral albumin uptake as a phenomenon not limited in subcutaneous allograft models. Consequently, PET imaging with [^89^Zr]­Zr-DFO*malHSA is a promising tool to image albumin accumulation in malignant tissue, which should be further developed toward clinical translation.

## 4 Experimental Section

### 4.1 General Remarks and Methods

Reagents and solvents, unless specified otherwise, were purchased from Sigma-Aldrich or VWR and used without further purification. DFO*maleimide was kindly provided by ABX advanced biomedical compounds GmbH and used without further purification. All radiolabeling buffers were prepared using Millipore water pretreated with Chelex© resin (Chelex© 100 sodium form (50–100 mesh, Sigma-Aldrich)), to remove trace metals, for 15 min and then filtered to remove the Chelex© beads (50 g L^–1^). All pipet tips and reaction tubes (Eppendorf) used for handling or storing protein samples were precoated with 0.1% Tween 80 in physiological saline (Braun). All compounds are >95% pure by size exclusion chromatography (SEC), iTLC, and spin filter analysis (chromatograms and iTLC of radiolabeled proteins are included in the Supporting Information).

#### 4.1.1 Protein Concentration

Quantification of protein concentration was determined using a microvolume UV/vis spectrophotometer (NanoDrop One^C^, Thermo Fisher Scientific). All measurements were performed in triplicates after a blank measurement with the respective buffer matrix of the to be measured protein solution. 1.5–2.0 μL sample were applied with a piston-operated pipet (Eppendorf Research plus, 0.1–2.5 μL, Eppendorf). The protein concentration was calculated by the measured absorbance at λ = 280 nm, HSA molecular weight of 66,437 Da and the molar extinction coefficient of ε = 35 495 M^–1^cm^–1^.^65^

#### 4.1.2 γ Counter

γ-counting was performed on a 2480 Wizard^2^ 1‑Detector γ counter with an energy window of 500–1000 keV (crystal: NaI (Tl), 80 mm in height, 75 mm in diameter; PerkinElmer). Counts were corrected for background radiation, physical decay, and counter dead time.

#### 4.1.3 Dose Calibrator

Sample radioactivity was measured with a VDC-405 dose calibrator V3.26 (Veenstra) calibrated for ^89^Zr.

#### 4.1.4 SEC

SEC was performed on an NGC Quest 10 Plus Chromatography System (Bio-Rad), with integrated UV detector for 4 wavelengths and conductivity unit, additionally equipped with a HERM LB 500 NaI γ detector (Berthold). Protein samples were eluted on a BioSep SEC-s2000 size-exclusion column (Phenomenex) with PBS buffer (pH 7.4, Mephisto) at a flow rate of 1 mL min^–1^. The freshly prepared buffer was degassed in an ultrasound bath for 30 min prior to all measurements.

#### 4.1.5 ITLC

iTLC was performed by spotting minimum 2 μL of sample on silica gel impregnated paper (Agilent) with 50 mM EDTA solution as mobile phase. After the development of the TLC plate, it was dried with a heating gun and the radioactivity on the plate was measured with a miniGITA dual device (Elysia-raytest) with 2 mm collimator height, detecting 500–1000 keV (measuring time: 1–8 min).

#### 4.1.6 Spin Filter Analysis

Radiochemical purity was determined as described previously.^42^ 10 μL of radiolabeling solution were diluted to 100 μL with formulation buffer (0.9% NaCl) and 5% DMSO. The solution was pipetted into a centrifugal filter unit (Microcon© Centrifugal Filter Unit with Ultracel-30 membrane and 30 kDa MWCO) which was subsequently centrifuged at 14,000*g* for 7 min. Afterward, the filter was washed two times with each 100 μL of washing buffer, that consisted of the formulation buffer and 5% DMSO. The combined filtrates and the filter were counted separately in a γ-counter and corrected for background and decay. The radiochemical purity was determined as the ratio of counts detected for the filter to the total number of counts. Unspecific binding was determined by adding [^89^Zr]­Zr^4+^ or radiolabeled DFO*mal to the spin filter and applying the same method.

#### 4.1.7 Coating of Disposable Plastics to Minimize Protein Retention

Preliminary experiments showed that at low protein concentration (<0.5 mg mL^–1^), the unspecific binding to disposable plastics (pipet tips, tubes, well-plates) was significant and varied, even for protein-low-binding pipet tips and tubes. Therefore, when handling low protein concentrations, all disposable plastics (except well-plates) were coated with a solution of 0.1% Tween 80 in 0.9% NaCl. For pipet tips, the Tween 80 solution was pipetted up and down five times, the tip was emptied, and the desired volume of protein solution was pipetted with the coated tip. Tubes were filled with Tween 80 solution and emptied after 10 s.

### 4.2 Characterization of Different Commercial HSA

Four different commercially available human serum albumins were evaluated for their suitability to be used for the synthesis of the radiotracer [^89^Zr]­Zr-DFO*malHSA. One albumin from human serum, purchased as pharmaceutical solution (Albunorm© (HSA1), Octapharma), two albumins of human serum, purchased as lyophilized powders (A1693 (HSA2) and A3782 (HSA3), Sigma-Aldrich), and one recombinant, lyophilized albumin (A9731 (HSA4), Sigma-Aldrich) were evaluated for their content of free thiols (DTNB assay), presence of aggregates according to SEC (UV trace at λ = 280 nm) and their uniformity by ESI-MS spectra (M^+^ and width of observed range of signals).

#### 4.2.1 DTNB Assay

DTNB assays were performed as described previously.^66^ Briefly, the protein was added to 0.1 M NaH_2_PO_4_/1 mM EDTA buffer (pH 8) in a quartz crystal cuvette equipped with a stirring bar to yield a final protein concentration of 80 μM. 50 μL of freshly prepared DTNB stock solution (10 mM in H_2_O) were added to yield a final volume of 1.25 mL. The solution was stirred for 20 min and the absorbance was measured at λ = 412 nm using a Thermo Fisher Genesys 180 UV–visible spectrophotometer. The protein blank (*A*_p_) was measured using buffer and protein in the absence of reagent, and the reagent blank (*A*_r_) was measured using buffer and reagent in the absence of protein. The reaction was performed in triplicates. The thiol concentration was calculated as [SH] = (*A*_412s_ – *A*_412r_ – *A*_412p_)/(ε_412_ × 1 cm) with Δε_412_ = 14 150 M^–1^ cm^–1^.^67^ The results were expressed as [SH]/[HSA] = % free thiol.

#### 4.2.2 Mass Spectrometry

Protein samples were reformulated in water via PD-10 columns, according to manufacturer’s instructions. The resulting protein solution was then lyophilized. A minuscule amount of the resulting powder was dissolved in 20 μL of solvent (2% ACN/98% H_2_O) and diluted 1:20. This was then further diluted 10-fold, and all dilutions were measured, and the best mass spectra was used for analysis.

Mass spectra were obtained using LTQ Orbitrap Velos mass spectrometer (Thermo Fisher Scientific, Bremen, Germany) equipped with a nanospray ion source, coupled to the nano HPLC-system (UltiMate 3000, Dionex). Five μL of the sample was first loaded on a μ-precolumn (trap column) PepMap300 (Thermo Scientific, 300 μm i.d. x 5 mm, C4, 5 μm, 300 Å) using (2% ACN, 98% H_2_O, 0.1% TFA) at a flow rate of 10 μL min^–1^. Separation was carried out on a C4 analytical column Accucore (Thermo Scientific, 75 μm x 50 cm, nanoViper C4, 2.6 μm, 150 Å) at a flow rate of 300 nL min^–1^. Column Oven Temperature = 40.0 °C. Mobile phase A: (2% ACN, 98% H_2_O, 0.1% FA). Mobile phase B: (80% ACN, 20% H_2_O, 0.1% FA). Gradient: 0 min-2% B, 5 min-5% B, 6 min-10% B, 20 min-60% B, 21 min-80% B, 25 min-80% B, 26 min-2% B, 30 min-2% B. Full-scan mass spectra were acquired in positive ion mode at 400–2500 *m*/*z* range at a resolution of 7500. The electrospray voltage was 2.1 kV and the ion transfer capillary temperature was 300 °C. Data were processed with Thermo Xcalibur 2.2 SP1.48 Qual Browser. Charge state determination and deconvolution of electrospray ionization mass-to-charge ratio spectra and determination of the molecular mass of DFO*malHSA was performed using the MagTran software.^68^

### 4.3 Synthesis of DFO*malHSA

Albunorm© (HSA1, 200 mg mL^–1^, Octapharma) was reformulated in PBS buffer (pH 7.4) via PD-10 column in order to remove the pharmaceutical formulation buffer. The PD-10 column was equilibrated with at least 25 mL PBS. Then, 1 mL Albunorm© was loaded onto the column. 1.5 mL PBS were loaded, and the eluted buffer was discarded. HSA was then eluted with 3 mL PBS. Concentration was determined using microvolume UV/vis. SEC confirmed removal of residual buffer at λ = 220 nm and protein purity at λ = 280 nm. Aliquots were kept at −20 °C.

DFO*mal (1.03 mg, 1129 nmol, 1.5 equiv) was dissolved in 50 μL DMSO at 37 °C. HSA (50 mg, PBS, pH 7.4) was added and the reaction volume was adjusted with PBS to a maximum concentration of DMSO of 5%. If DFO*mal was not dissolved completely in DMSO or added to PBS (instead of PBS to DMSO), precipitation occurred which could not be reversed under mild conditions. The reaction mixture was gently shaken (300 rpm) at 37 °C for 45 min. 25 μL of an aqueous 40 mM *N*-acetyl-cysteine solution were added to quench remaining maleimide. After 10 min, the reaction was reformulated to 0.5 M 4-(2-hydroxyethyl)-1-piperazineethanesulfonic acid (HEPES) buffer (pH 7.4) using a PD-10 column according to manufacturer’s instructions. The conjugate mixture was eluted in 3 mL HEPES buffer. Afterward, it was washed three times using an Amicon© 4 centrifugal spin filter (30 kDa MWCO). The filter was spun at 4000*g* for 15 min at 20 °C. Before each centrifugation, the total volume in the filter was adjusted to 4 mL with HEPES buffer. The bioconjugate was analyzed by SEC and the protein concentration was determined via microvolume UV/vis. Aliquots were kept at −20 °C. MS analysis was performed for three different batches.

### 4.4 Radiolabeling of DFO*malHSA

#### 4.4.1 Radiolabeling of DFO*malHSA–Low Specific Activity

A frozen aliquot of 2 mg DFO*malHSA (100 μL, 0.5 M HEPES, pH 7.4) was thawed at 37 °C. 0.5–20 MBq [^89^Zr]­Zr^4+^ in 1 M oxalic acid (PerkinElmer) were put into an Eppendorf tube and the volume was adjusted to 50 μL with 1 M oxalic acid. The solution was neutralized with 22.5 μL 2 M Na_2_CO_3_. 327 μL HEPES buffer were added, followed by the DFO*malHSA solution. After 2 h at 37 °C, iTLC showed the percentage of free [^89^Zr]­Zr^4+^ and the RCP was determined. Thirty μL of 25 mg mL^–1^ EDTA solution were added to the reaction, and after 15 min the crude radiotracer was purified and reformulated in 3 mL 0.9% NaCl via PD-10 column. As purification with PD-10 columns only was not sufficient to obtain a pure product, Amicon© 4 centrifugal spin filters were used additionally. The solution was pipetted into the spin filter, and the volume was filled to 4 mL with 0.9% NaCl. The filter was spun at 4000*g* for 15 min at 20 °C. Afterward, it was washed two more times with each 4 mL 0.9% NaCl at 4000*g* for 15 min at 20 °C. When using spin filtration only, more than five centrifugation cycles were required to achieve >99% RCP. To minimize radiation exposure caused by multiple centrifugation steps using an unshielded centrifuge, the efficient combination of both purification methods as described above was pursued. The purified radiotracer was then removed from the filter and the total radioactivity was determined via dose calibrator. [^89^Zr]­ZrDFO*malHSA was analyzed by SEC, ITLC, spin filter analysis and microvolume UV/vis. Apparent specific activity was calculated by the radioactivity measured in a defined volume of the radiotracer solution after purification, and the protein concentration measured by microvolume UV/vis.

#### 4.4.2 Radiolabeling of DFO*malHSA–High Specific Activity

A frozen aliquot of 0.5–1 mg DFO*malHSA (200 μL in 0.5 M HEPES, pH 7.4) were thawed at 37 °C. 70.6 – 160.4 MBq [^89^Zr]­Zr^4+^ in 1 M oxalic acid (PerkinElmer) were transferred into an Eppendorf tube and the volume was adjusted to 100 μL with 1 M oxalic acid. The solution was neutralized with 45 μL 2 M Na_2_CO_3_. 633 μL HEPES buffer were added, followed by the DFO*malHSA solution. The reaction was left at 37 °C overnight to maximize the RCY and specific activity, as well to facilitate the workflow of subsequent biological experiments. After approximately 12 h, iTLC showed the percentage of free [^89^Zr]­Zr^4+^ and the RCP was determined. 63 μL of 25 mg mL^–1^ EDTA solution were added, and after 15 min the reaction was purified, reformulated and quality control was performed as described above.

### 4.5 Stability of [^89^Zr]­Zr-DFO*malHSA

#### 4.5.1 Stability in 0.9% NaCl and Human Blood Serum

To 1 MBq of [^89^Zr]­Zr-DFO*malHSA 0.9% NaCl was added to yield a total volume of 100 μL. The solution was added to 900 μL of 0.9% NaCl or human blood serum (Dunn Labortechnik), respectively. The solutions were kept at 37 °C over the course of 7 days (*n* = 3). Samples were taken after 30 min, 1, 2, 3, and 7 days and analyzed for their RCP via spin filter analysis and iTLC.

#### 4.5.2 Stability of [^89^Zr]­Zr-DFO*malHSA for Cell Uptake Experiments

To 1 MBq of [^89^Zr]­Zr-DFO*malHSA 0.9% NaCl was added to yield a total volume of 100 μL. The solution was added to 900 μL of the respective solution, buffer, or cell culture medium. Tested were 0.9% NaCl, human blood serum and HBSS with MES buffer. The solutions were kept at 37 °C over the course of 4 h. Samples were taken after 30 min, 1, 2, and 4 h and analyzed for their RCP via spin filter analysis.

### 4.6 Cell Culture

For the *in vitro* experiments, the human colorectal cancer model SW480 and the murine (Balb/c) colorectal cancer model CT26 were used and grown, unless otherwise stated, in either Minimum Essential Medium (MEM) or Dulbecco’s Modified Eagle Medium/Nutrient Mixture F-12 (DMEM/F-12) (1:1) medium, respectively. Both cell lines were purchased from American Type Culture Collection (ATCC, Rockville, MD). The cells were grown in a humidified incubator (37 °C, 5% CO_2_) in the above-mentioned medium containing 10% FCS (PAA, Linz, Austria). All cells were cultivated without the addition of antibiotics and were regularly checked for mycoplasma contamination.

#### 4.6.1 Cellular Uptake of FITC-Labeled HSA

The cells were plated in 12-well plates (2.5 × 10^5^ cells ml^–1^ well^–1^) and left to recover for 24 h. Followed by 24 h serum starvation by exchanging the FCS-containing medium with a serum-free RPMI medium. FITC-labeled HSA (MP chemicals) (5 μM) were diluted in HBSS (with Ca^2+^ and Mg^2+^) (Sartorius) containing 0.7% 1 M MES (pH 6 ± 0.05) and added to the cells which were incubated either at 37 or 4 °C to verify between an active (energy-dependent) and a passive (energy-independent) cellular uptake, respectively. After 3 h, the cells were harvested by trypsinization, pellets were washed (three times), diluted in PBS and the fluorescence was measured by flow cytometry (excitation: λ = 488 nm and emission: λ = 530 nm) using a BD Fortesssa flow cytometer (BD Bioscience). Further data processing was done by FlowJo software V10 (BD Bioscience).

#### 4.6.2 Cellular Uptake of [^89^Zr]­Zr-DFO*malHSA

SW480 cells were plated in 12-well plates (2.5 × 10^5^ cells ml^–1^ well^–1^) and left to recover for 24 h. Followed by 24 h serum starvation by exchanging the FCS-containing medium with a serum-free RPMI medium. Before the addition of 100 μL [^89^Zr]­Zr-DFO*malHSA solution (0.01, 0.05, 0.1, 0.5, 1, and 10 nm in 0.9% NaCl), the cells were washed with DPBS, and 900 μL HBSS (with Ca^2+^ and Mg^2+^) containing MES were added. Each concentration was incubated in triplicates for 3 h at 37 °C and 5% CO_2_ atmosphere. The supernatant was collected in a tube together with the two times 1 mL DPBS used for washing the cells. The cells were then lysed with 1 mL 1 M NaOH solution at 37 °C. The solution was collected in a separate tube together with the two times 1 mL DPBS used for washing the wells. The separated fractions from cells and supernatant were measured in a γ-counter, the measured counts were decay and background corrected, and the percentage of internalized radioactivity was calculated as fraction of internalized versus total radioactivity.

### 4.7 Animal Experiments

#### 4.7.1 Animals and CT26 Allograft

Eight- to 12-week-old female Balb/c mice (*n* = 20) were purchased from Envigo Laboratories (San Pietro al Natisone, Italy). The animals were kept in a pathogen-free environment, with 12 h light–dark cycle and every procedure was done in a laminar airflow cabinet. Tumor growth was evaluated by daily recording of tumor size by caliper measurement. Tumor volumes (mm^3^) were calculated using the formula: (length × width^2^)/2. All experiments were approved by the Ethics Committee for the Care and Use of Laboratory Animals at the Medical University Vienna (proposal number BMWF 2022–0770–291) and performed according to the guidelines from the Austrian Animal Science Association and from the Federation of European Laboratory Animal Science Associations (FELASA).

For the allografts, CT26 cells (5 × 10^5^ in 50 μL serum-free RPMI medium) were subcutaneously injected into the right flank of female Balb/c mice (*n* = 31). After 8 d of tumor growth, animals were randomized and used for experiments (biodistribution or PET imaging). Additionally, F-luciferase-transfected CT26 cells (1 × 10^5^ in 100 μL serum-free RPMI medium)^69^ were intraperitoneally injected into one animal. After 8 days of tumor injection, this animal received [^89^Zr]­Zr-DFO*malHSA (see 4.7.3) and was imaged by PET/CT for one time point (48 h p.i.). Additionally, it received bioluminescence imaging post-mortem (see 4.7.6).

#### 4.7.3 PET/CT Measurements and Ex Vivo Biodistribution

Mice bearing CT-26 subcutaneous allografts (*n* = 7, mean tumor volume 300 mm^3^ determined via caliper measurement) or an intraperitoneal CT26 allograft (*n* = 1) were anesthetized using isoflurane (2.5% in 1.5 mL oxygen) and intravenously injected with 8.02 ± 2.9 MBq [^89^Zr]­Zr-DFO*malHSA via a tail vein. Injection volumes never exceeded 150 μL per i.v. application. Animals were kept under anesthesia for 30 min or returned to their cages for imaging and/or organ removal at later time points (24, 48 and 72 h). Animals were placed into the field of view of the scanner (μPET/CT Inveon, Siemens Medical Solution, Knoxville), and a 7 min cone beam CT was performed followed by a static PET acquisition for 10 min. Vital parameters (respiration, body temperature) were continuously monitored using a dedicated monitoring unit (bioVet; m2m imaging, Cleveland, OH) to ensure the depth of anesthesia and wellbeing of the animals. PET images were reconstructed using OSEM3D, corrected for scatter, attenuation (generated from CT images) and decay (image data were corrected for PET acquisition start). Rigid-body image registration and image quantification was performed using the image analysis software PMOD 3.8 (PMOD Technologies Ltd., Zurich, Switzerland) and Inveon Research Workplace (IRW; Siemens Medical Solutions, Knoxville). Volumes of interest (VOI) were delineated manually and kBq/cc were extracted for each VOI. Percentage of injected dose per cm^3^ (%ID/cc) and standardized uptake values (SUV, normalized for body weight) were subsequently calculated. PET/CT imaging was performed in seven animals, with four of the animals being measured again at a later time point. All animals were sacrificed at the end of imaging experiments and collected organs and tissues were measured in a γ-counter.

#### 4.7.4 Ex Vivo Biodistribution and Plasma Stability of [^89^Zr]­Zr-DFO*malHSA in Murine Blood Serum

In addition to the seven animals that were used for PET/CT imaging, 12 animals were injected with 0.75 ± 0.5 MBq [^89^Zr]­Zr-DFO*malHSA and sacrificed by cervical dislocation at different time points (30 min, 24 h, 48 h and 72 h p.i., *n* = 3 per time point). Organs were harvested, weighed and measured in a γ-counter. Counts per minute were decay corrected and normalized to weight and injected dose and expressed as the percentage injected dose per gram of tissue (%ID g^–1^). Statistical analyses (paired or unpaired *t* test) were performed using GraphPad Prism software version 8.2.1 (GraphPad Software, Inc., San Diego, CA). Data with a *p*-value <0.05 was considered significant.

Blood samples were taken directly after scarification and collected in Tween 80 coated 1.5 mL tubes. Samples were weighted and the radioactivity was measured in a γ-counter, analogous to all other biodistribution samples. Afterward, the murine blood serum was obtained by centrifugation at 1000*g* for 10 min at 4 °C. The serum was transferred to another tube. The remaining blood pellet was washed by adding 200 μL 0.9% NaCl and centrifuging the mixture again at 1000*g* for 10 min at 4 °C. The supernatant was transferred to the serum containing tube. The radioactivity of the combined serum fraction and the blood pellet was measured in a γ-counter. 10 μL of the serum fraction were subjected to spin filter analysis in order to determine the RCP of the radiotracer in murine blood serum *ex vivo*.

#### 4.7.5 Cellular Uptake of Radioactivity in Ex Vivo Tumors

The tumor tissue of the mice sacrificed 48 and 72 h p.i. (*n* = 2 each, *n* = 4 total) was further analyzed for the radioactivity present in cellular fractions versus extracellular tumor tissue. CT26 tumors were dissected, and after weighing and radioactivity measurements of the whole tumor, specimens were cut into approximately 1 mm^3^ pieces on a Petri dish using a scalpel. The tumor pieces were transferred to a 15 mL Falcon tube, 2.35 mL RPMI-1640 medium were added, and the tissue was enzymatically digested with MACS tumor dissociation kit (Miltenyi Biotec) for 1 h at 37 °C, according to the manufacturer’s instructions. After filtering using a 70 mm cell strainer and washing with 10 mL PBS containing 1% FCS, the suspended cells were centrifuged at 400*g* for 3 min at 4 °C. After removing and collecting the supernatant in a separate tube, the pelleted cells were suspended in 1 mL red blood cell lysis buffer and incubated on ice for 3 min to lyse red blood cells. After the addition of 5 mL PBS containing 1% FCS, the suspended cells were centrifuged at 400*g* for 3 min at 4 °C. The supernatant was collected again in the same tube and the cell pellet was washed with 4 mL PBS containing 1% FCS, the suspended cells were centrifuged at 400*g* for 3 min at 4 °C and the supernatant was collected again in the same tube. Radioactivity of both the cell pellet and the supernatants fractions was determined in a γ-counter. Cells were counted and their viability was verified.

#### 4.7.6 HSA Distribution in CT-26-Bearing Balb/c Mice by Immunohistological Analysis

Mice bearing CT26 subcutaneous allografts (*n* = 12, mean tumor volume 250 mm^3^ determined via caliper measurement) were treated with 1 g/kg HSA (HSA1, Albunorm 20%) i.v. After 30 min, 24, 48 and 72 h animals (*n* = 3 per time point) were sacrificed by cervical dislocation and tumor and organs (liver, kidney, lung, spleen, colon, pancreas and brain) were collected. All organs were fixed in 4% formaldehyde for 24 h (Carl Roth, # P087.3), dehydrated and paraffin-embedded. For evaluation of the HSA content in CT26 tumors and organs, the tissues were sliced in 3.5 μm thick sections, deparaffinized and rehydrated. Subsequently, the samples were heated for 10 min in 10 mM citrate buffer (pH 6.0) followed by incubation with the HSA-specific HRP-conjugated primary antibody (A80–129P, Biomol: dilution 1:200 at 4 °C overnight) diluted in 0.1% Tween 20 and 1% goat serum-containing PBS. Binding of primary antibodies was detected by incubation with 3,3-diaminobenzidine (Dako, K3468) and counterstaining with hematoxylin Gill III (Merck, 1.05174.0500). HSA specificity was proven using tissues form CT26 bearing non-HSA-treated Balb/c mice (Figure S24). Quantitative analysis of the % of HSA-positive areas was achieved by HALO software.

#### 4.7.7 Bioluminescence Imaging

Ten days after tumor inoculation, orthotopic tumors were imaged by bioluminescence imaging (BLI) using an *in vivo* imaging system (IVIS Spectrum, PerkinElmer). The mouse was injected with approximately 200 μL of a 15 mg mL^–1^ solution of D-luciferin (150 mg kg^–1^ body weight, IVISbrite, PerkinElmer). After 15 min, animals were sacrificed and images of the whole mouse with opened peritoneum and single organs (heart, lung, pancreas, liver, spleen, four tumor samples) were acquired (exposure time 3 s, binning factor 8, field-of-view 13.2 cm, f/stop 1, no filter).

## Supplementary Material





## Data Availability

The data that support the findings of this study are available from the corresponding author upon reasonable request.

## Notes

Molecular formula string (CSV)
SEC chromatograms & MS data of different HSA (Figure S1–S8); SEC chromatograms and MS data of bioconjugation (Figure S9–S11); iTLC (Figure S12); SEC chromatograms of radiolabelings (Figure S13–S17); cellular uptake of FITC-labeled and radiolabeled HSA (Figure S18 – S20); PET MIP fused with CT of all animals (Figure S21); PET image quantification (Figure S22 and S23); Immunohistological analysis of HSA in mice (Figure S24–S26); post-mortem and *ex vivo* organ bioluminescence imaging of orthotopic tumor model (Figure S27); biodistribution data (Table S1–S4) (PDF)

## Abbreviations Used

ACN: acetonitrile
BLI: bioluminescence imaging
cc: cubic centimeter
CRC: colorectal cancer
CT: computed tomography
DFO: deferoxamine
DMEM: Dulbecco’s Modified Eagle Medium
DPBS: Dulbecco’s Phosphate Buffered Saline
DTNB: 5,5′-Dithiobis­(2-nitrobenzoic acid)
EPR: enhanced retention and permeability
FcRn: neonatal Fc receptor
FCS: fetal calf serum
FITC: fluorescein isothiocyanate
HBSS: Hanks’ Balanced Salt Solution
HEPES: 4-(2-hydroxyethyl)-1-piperazineethanesulfonic acid
ID: injected dose
ITLC: instant thin-layer chromatography
kDa: kilo Dalton
mal: maleimide
MEM: minimum essential medium
MES: 2-(*N*-morpholino)­ethanesulfonic acid
MIP: maximal intensity projection
MRI: magnetic resonance imaging
MWCO: molecular weight cutoff
RCP: radiochemical purity
RCY: radiochemical yield
RPMI: Roswell Park Memorial Institute
rt: retention time
SD: standard deviation
SEC: size-exclusion chromatography
VOI: volume of interest
