# Supplementary material for: Site-Selectively Functionalized Albumin with DFO*Maleimide for 89Zr-Radiolabeling Yields a Metabolically Stable PET Probe that Enables Late Time-Point Tumor Imaging in Mice
Source: J Med Chem. 2025 Jun 17;68(12):12925–39. doi: 10.1021/acs.jmedchem.5c00803 (PMC12207588; doi:10.1021/acs.jmedchem.5c00803)
Supplement: Supplementary file 2 [file jm5c00803_si_002.pdf]

Supporting Information

Site-selectively functionalized albumin with DFO*maleimide for 89Zr-radiolabelling yields a metabolically stable PET probe that enables late time-point tumor imaging in mice

Julia Kronberger, Theresa Balber, Hemma Schueffl, Raphaela Wahrmann, Anja Federa, Mathias Gradl, Marie R. Brandt, Thomas Wanek, Markus Mitterhauser, Christian R. Kowol, Thomas L. Mindt, Petra Heffeter.*

** Corresponding author, E-mail: thomas.mindt@univie.ac.at*

J. Kronberger, A. Federa, M. Brandt, M. Mitterhauser, C. R. Kowol, T. L. Mindt
Institute of Inorganic Chemistry, Faculty of Chemistry, University of Vienna, @, 1090 Vienna, Austria.

J. Kronberger, T. Balber, M. Brandt, M. Mitterhauser, T. L. Mindt
Ludwig Boltzmann Institute Applied Diagnostics, General Hospital of Vienna, Währinger Gürtel 18-20, 1090 Vienna, Austria.

J. Kronberger, A. Federa
Vienna Doctoral School in Chemistry, University of Vienna, Währinger Straße 42, 1090 Vienna, Austria.

T. Balber, M. Mitterhauser, T. L. Mindt
Department of Biomedical Imaging and Image Guided Therapy, Division for Nuclear Medicine, Medical University of Vienna, Währinger Gürtel 18-20, 1090 Vienna, Austria.

H. Schueffl, R. Wahrmann, M. Gradl, P. Heffeter
Center for Cancer Research and Comprehensive Cancer Center, Medical University of Vienna, Borschkegasse 8a, 1090 Vienna, Austria.

T. Balber, M. Brandt, M. Mitterhauser, T. L. Mindt
Joint Applied Medicinal Radiochemistry Facility, University of Vienna, Medical University of Vienna, 1090 Vienna, Austria.

T. Wanek
Department of Biomedical Imaging and Image Guided Therapy, Preclinical Imaging Laboratory (PIL), Medical University of Vienna, 1090 Vienna, Austria.

Table of contents

I. SEC Chromatograms of different HSA P3
II. MS Data of different HSA P5
III. SEC Chromatograms and MS data of Bioconjugation....................P7
IV. ITLC....................P8
V. SEC Chromatograms of Radiolabelings....................P9
VI. *In Vitro* Studies P11
VII. Animal Studies P13

I. SEC Chromatograms of different HSA

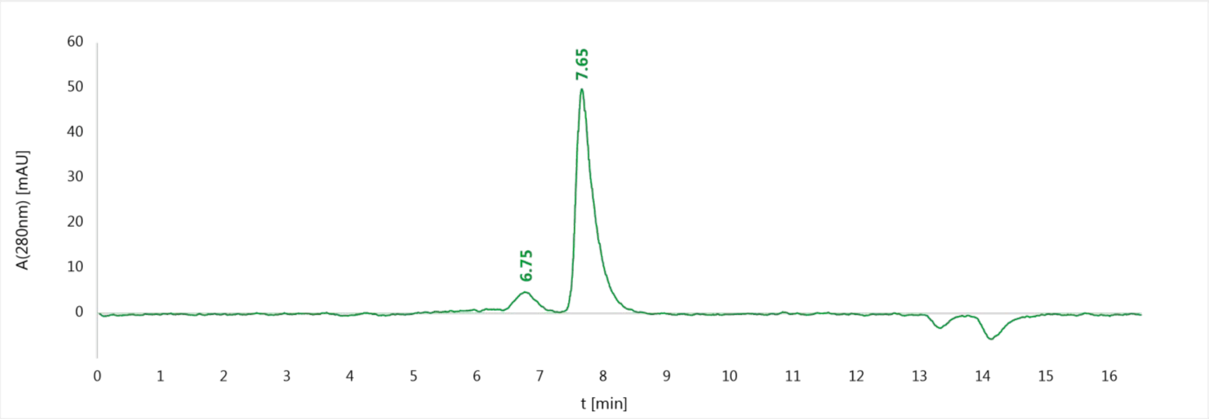

Figure S1. SEC chromatogram of A1653 (HSA2), UV signal at λ = 280 nm.

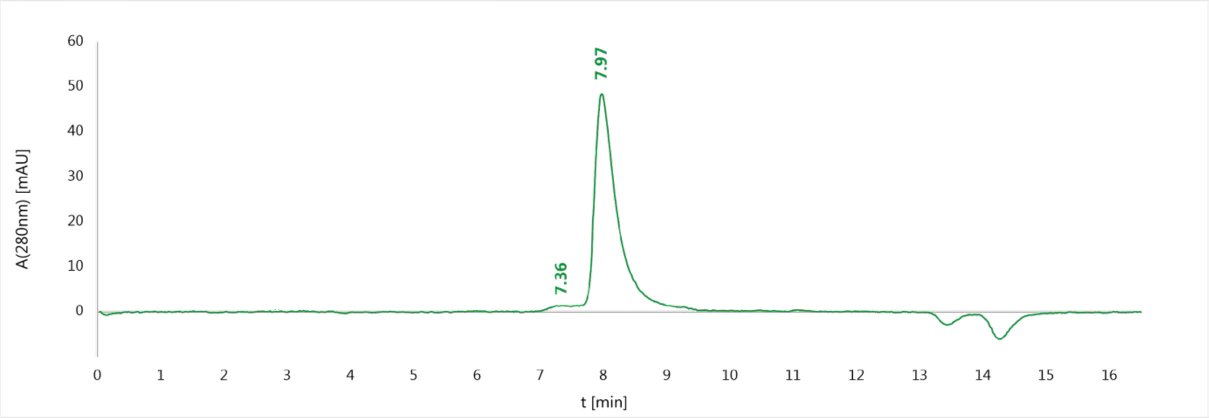

Figure S2. SEC chromatogram of A9731 (HSA4), UV signal at λ = 280 nm.

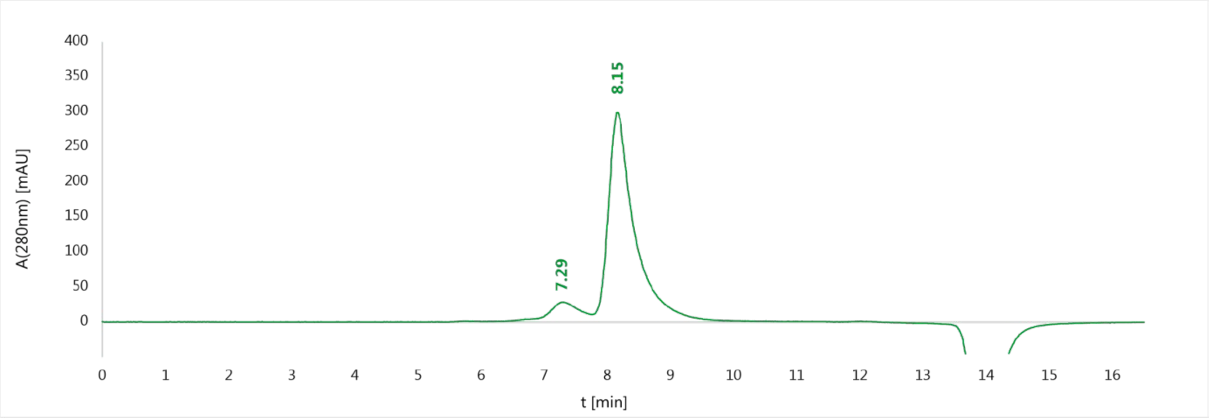

Figure S3. SEC chromatogram of A3782 (HSA3), UV signal at λ = 280 nm.

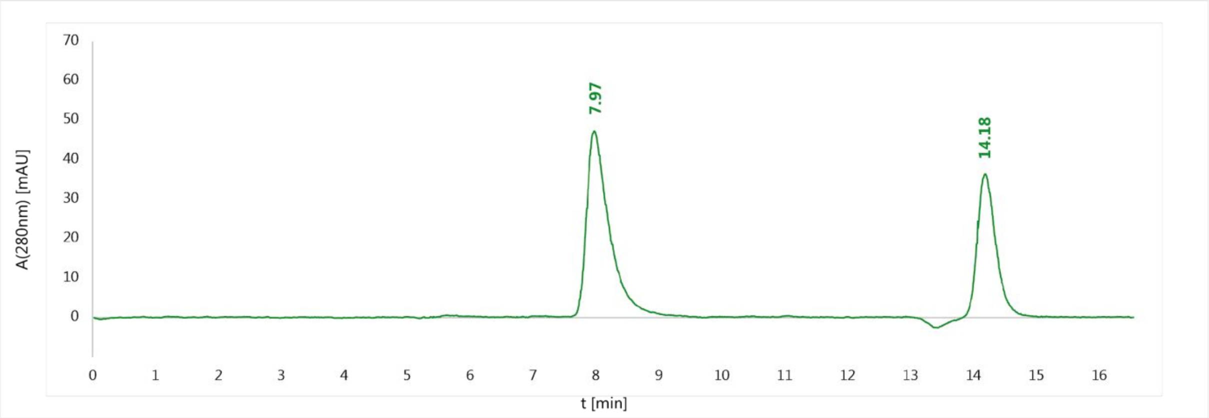

Figure S4. SEC chromatogram of Albunorm© (HSA1), UV signal at λ = 280 nm.

II. MS Data of different HSA

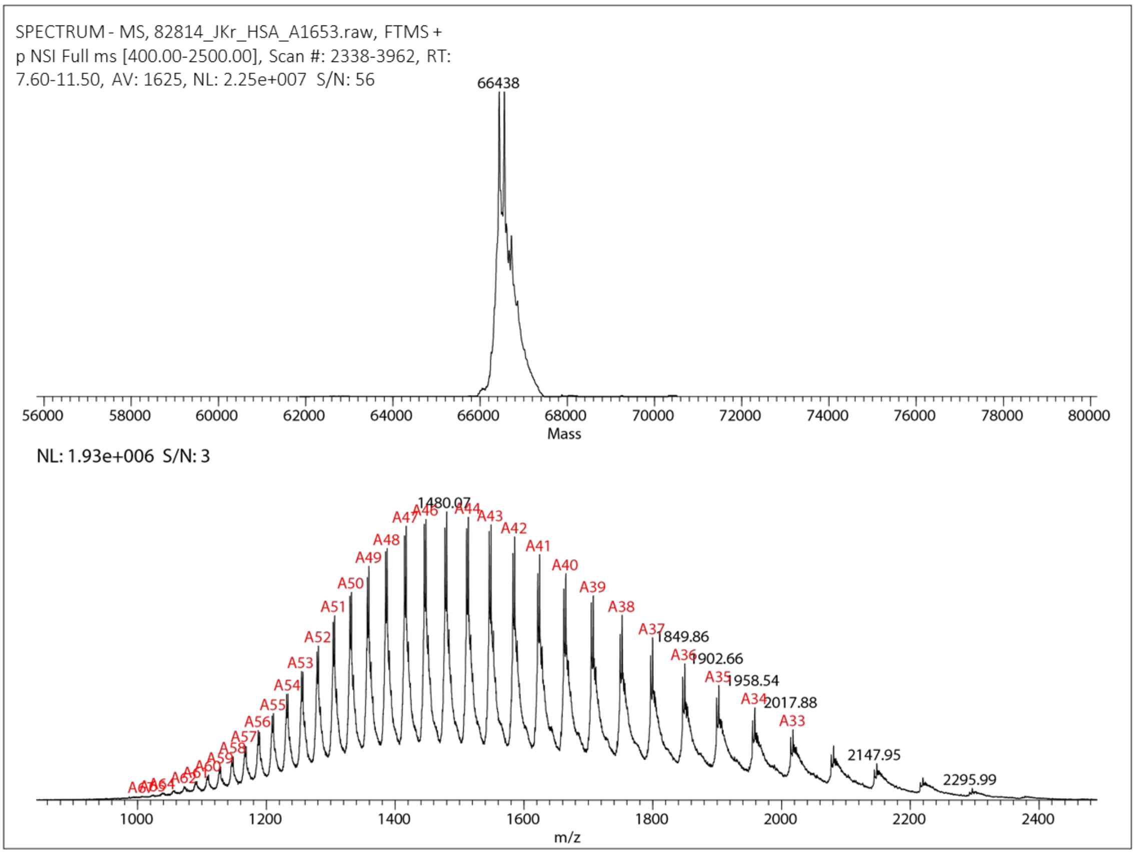

Figure S5. Deconvoluted ESI-MS spectrum of HSA A1653 (HSA2).

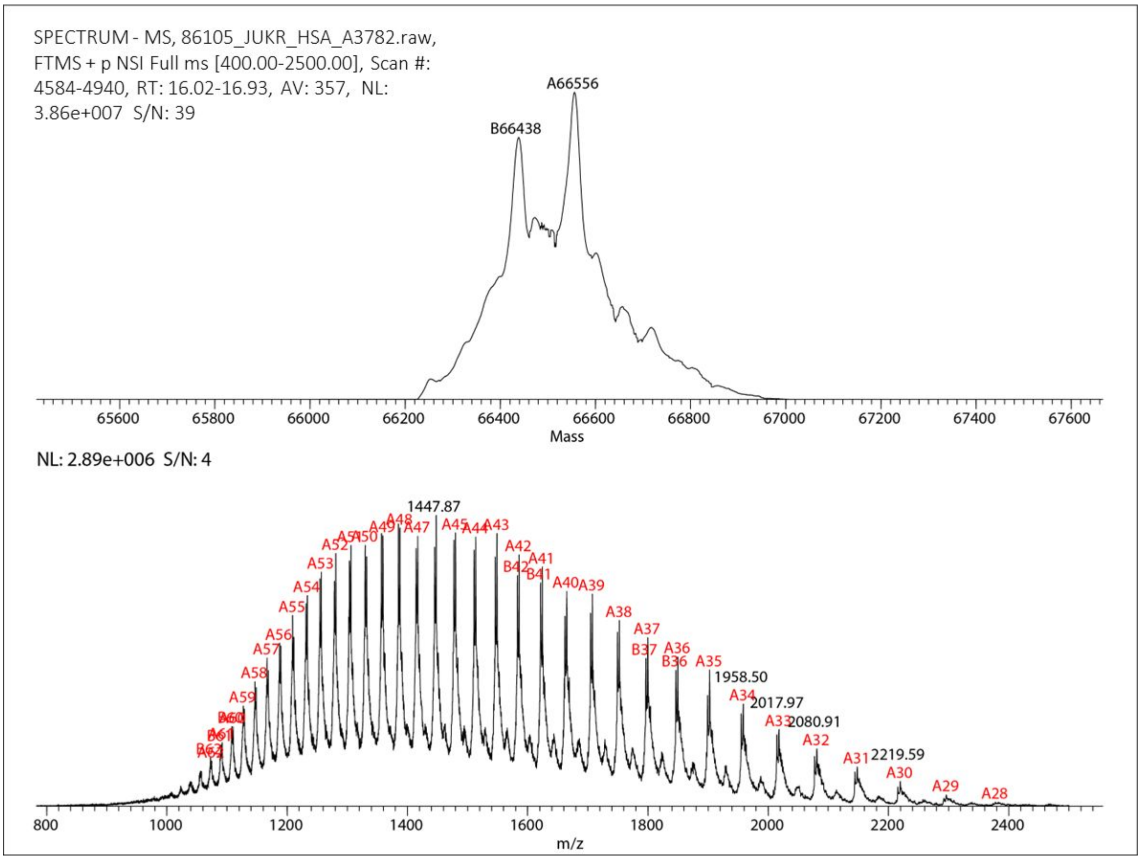

Figure S6. Deconvoluted ESI-MS spectrum of HSA A3782 (HSA3).

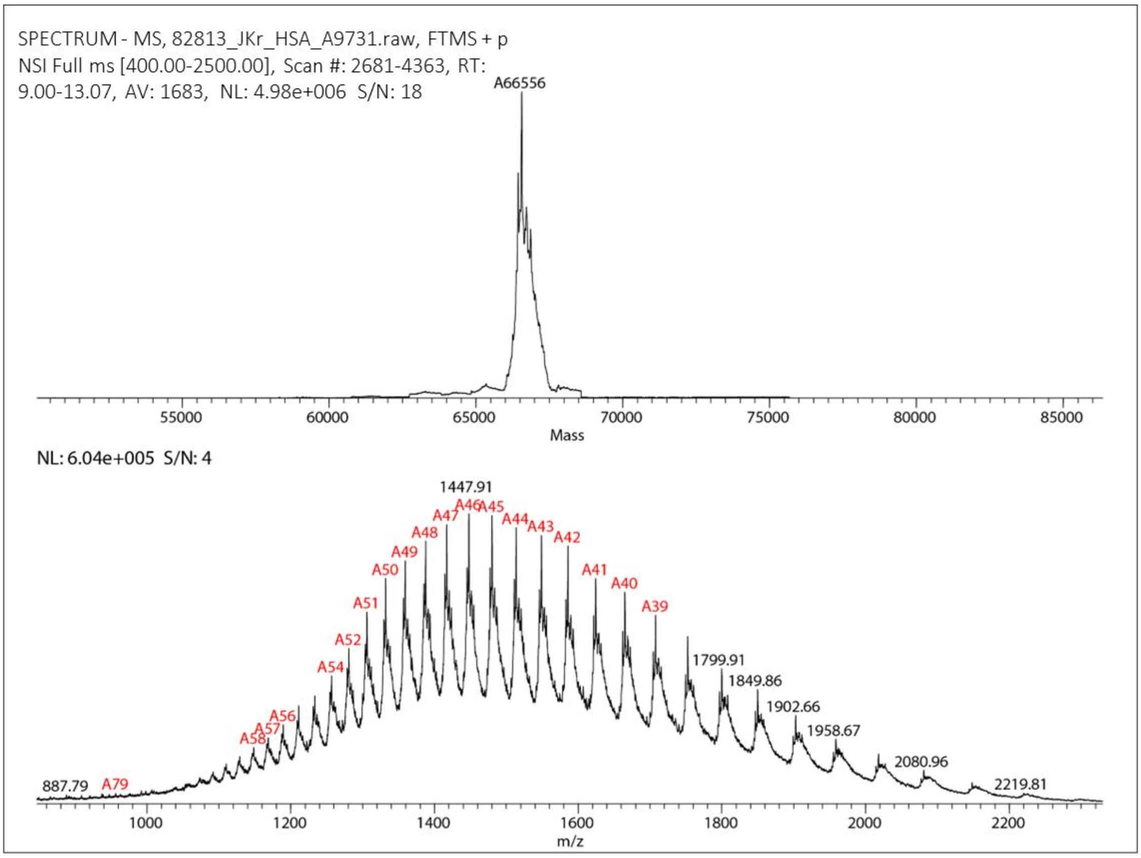

Figure S7. Deconvoluted ESI-MS spectrum of HSA A9731 (HSA4).

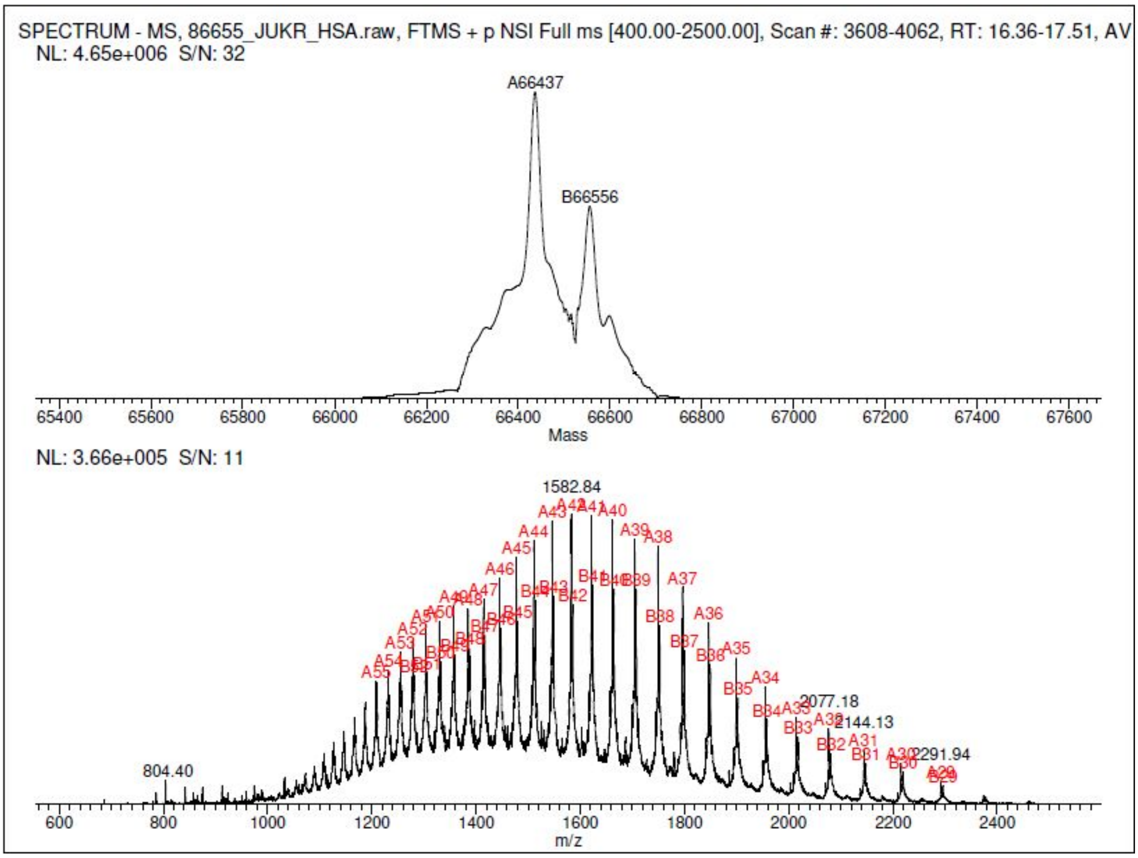

Figure S8. Deconvoluted ESI-MS spectrum of HSA Albunorm© (HSA1).

III. SEC Chromatograms and MS data of Bioconjugation

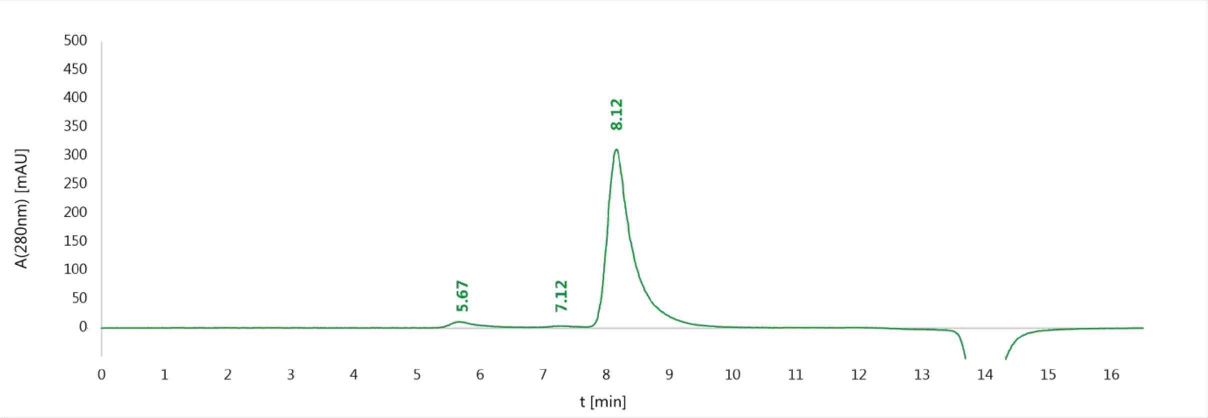

Figure S9. SEC chromatogram of desalted Albunorm© (HSA1).

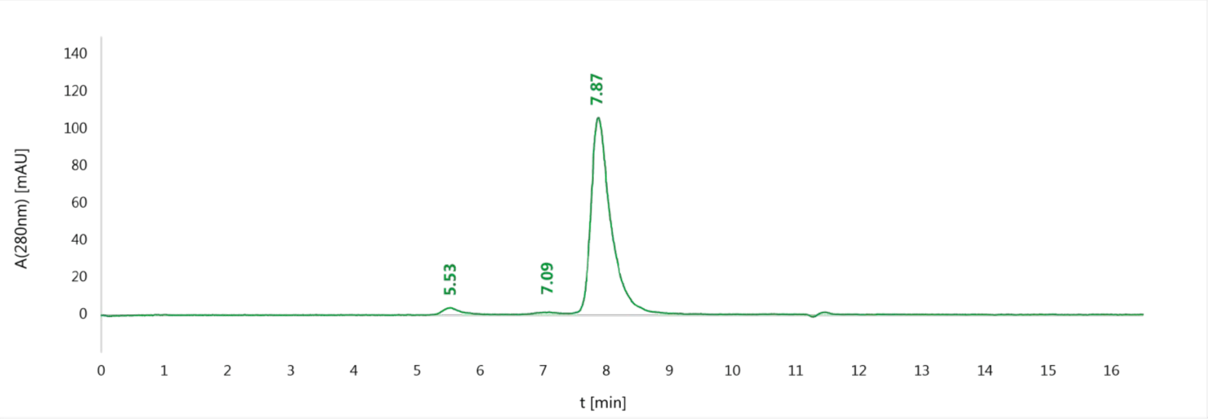

Figure S10. SEC chromatogram of DFO*malHSA.

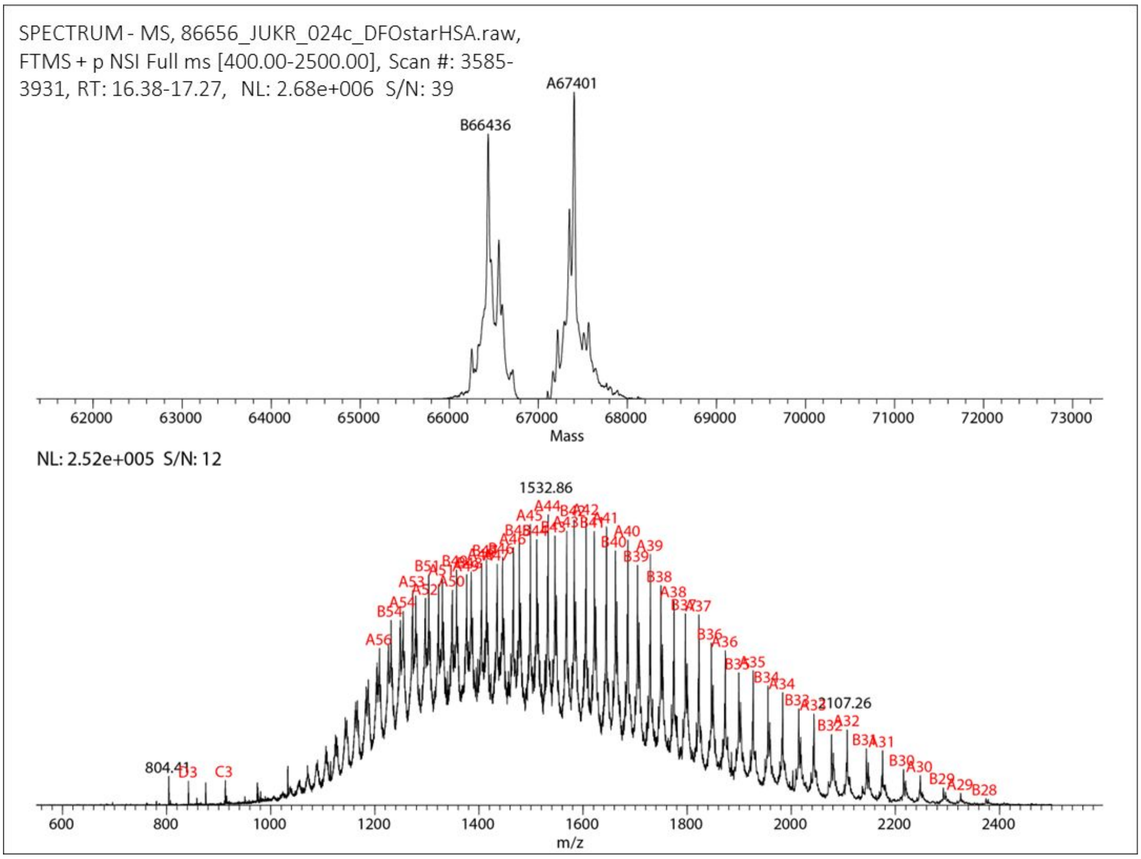

Figure S11. Deconvoluted ESI-MS spectrum of DFO*malHSA.

IV. ITLC

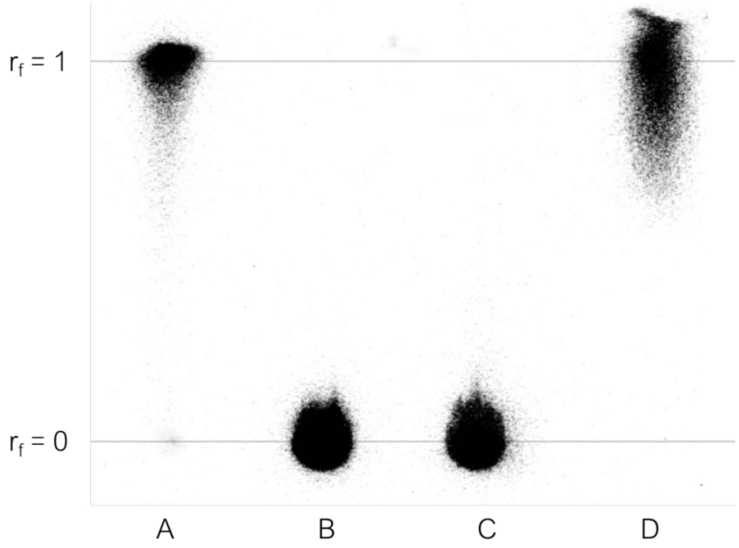

Figure S12: iTLC performed in 50mM EDTA on silica-impregnated chromatography paper of A) [^{89}Zr]Zr^{4+} in 1 M oxalic acid (free ^{89}Zr), B) [^{89}Zr]Zr^{4+} incubated with DFO*mal using radiolabeling conditions, C) [^{89}Zr]Zr-DFO*malHSA and D) HSA radiolabeled with [^{89}Zr]Zr^{4+} without chelator.

V. SEC Chromatograms of Radiolabelings

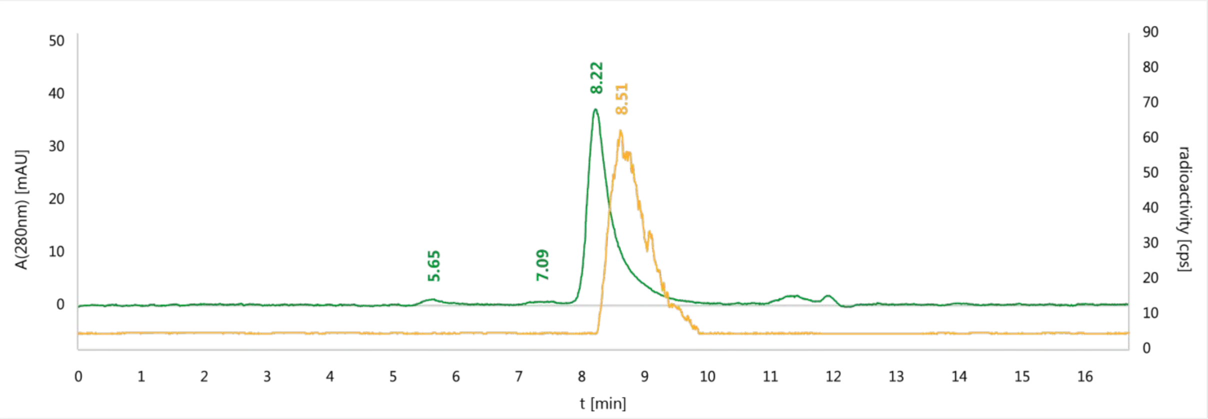

Figure S13. SEC chromatogram of crude [^{89}Zr]ZrDFO*malHSA with low specific activity (5.6-26.9 MBq mg^{-1}). Green trace = UV signal at λ = 280 nm, yellow = radiosignal. The small difference in retention time is caused by the serial arrangement of the two detectors.

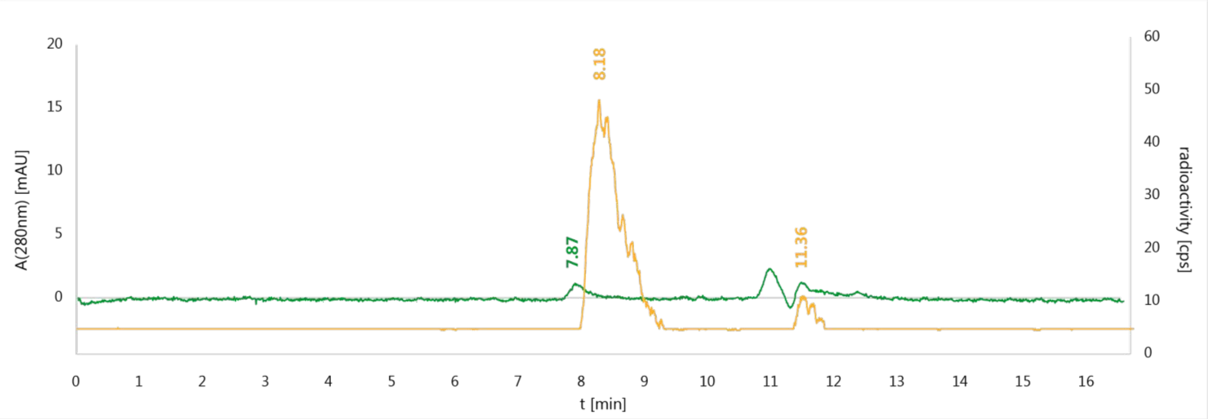

Figure S14. SEC chromatogram of crude [^{89}Zr]ZrDFO*malHSA with high specific activity (0.5 - 20 MBq mg^{-1}). Green trace = UV signal at λ = 280 nm, yellow = radiosignal. The small difference in retention time is caused by the serial arrangement of the two detectors.

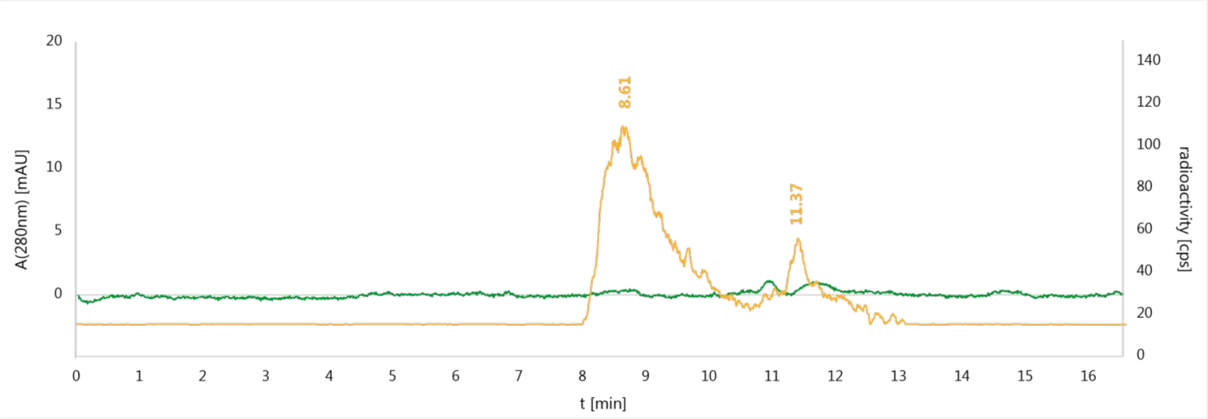

Figure S15. SEC chromatogram of PD-10 purified [^{89}Zr]ZrDFO*malHSA with high specific activity (up to 163.5 MBq mg^{-1}). Green trace = UV signal at λ = 280 nm, yellow = radiosignal. The small difference in retention time is caused by the serial arrangement of the two detectors.

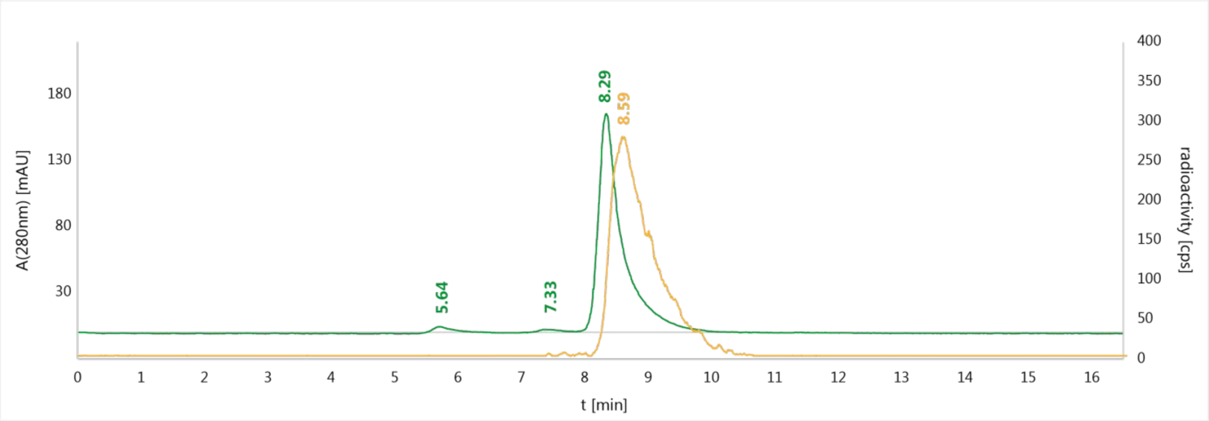

Figure S16. SEC chromatogram of purified [^{89}Zr]ZrDFO*malHSA with low specific activity (up to 163.5 MBq mg^{-1}). Green trace = UV signal at λ = 280 nm, yellow = radiosignal. The small difference in retention time is caused by the serial arrangement of the two detectors.

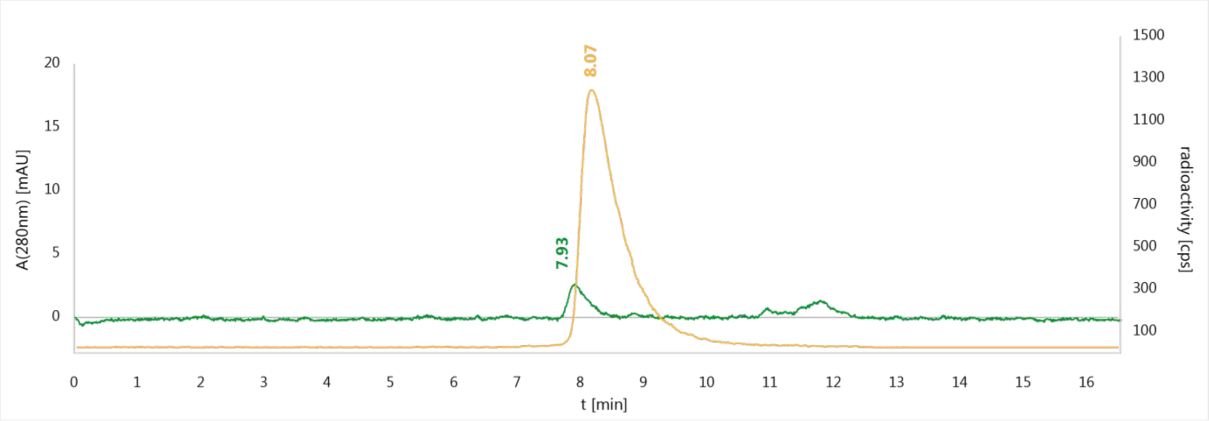

Figure S17. SEC chromatogram of purified [^{89}Zr]ZrDFO*malHSA with high specific activity (up to 163.5 MBq mg^{-1}). Green trace = UV signal at λ = 280 nm, yellow = radiosignal. The small difference in retention time is caused by the serial arrangement of the two detectors.

VI. *In Vitro* Studies

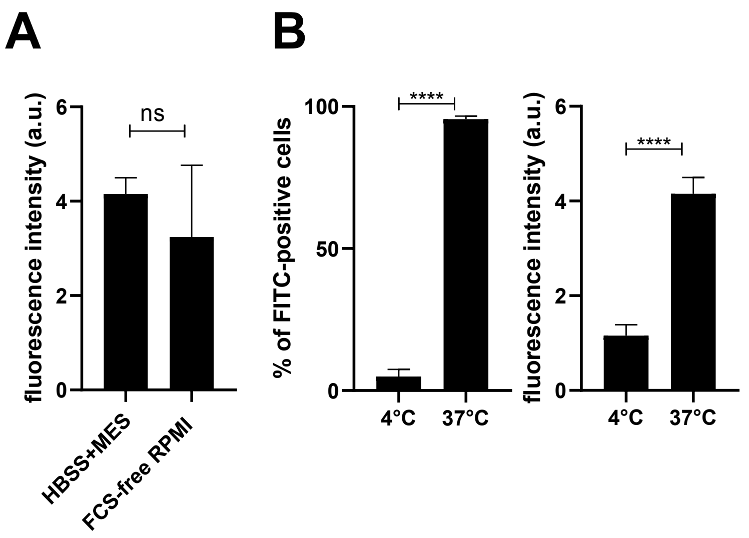

Figure S18: Cellular uptake of FITC-labelled HSA in the human colon cancer model SW480 after 3 h. Comparing the impact of (A) HBSS+MES with FCS-free RPMI medium as solvent as well as (B) the two incubation temperatures 37 °C and 4 °C. Fluorescence intensity was determined by flow cytometry and normalized to auto fluorescence control. Given values are mean +/- SD of 3-4 independent experiments. Statistical significance was tested by an unpaired two-tailed t-test (**** $p<0.0001$ and *** $p<0.001$, ns = not significant)

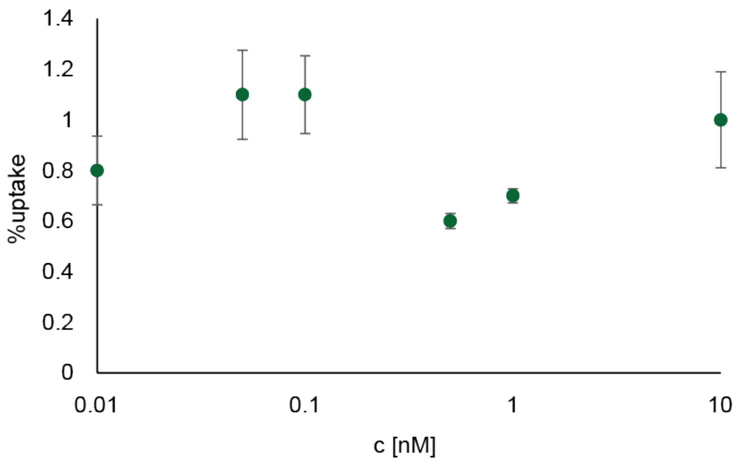

Figure S19 *In vitro* uptake of [^{89}Zr]Zr-DFO*malHSA in SW480 cells at 6 different tracer concentrations from 0.01 to 10 nM with specific activities of 17.5-64.7 MBq mg^{-1}. Uptake measured after 3 h of incubation at 37 °C and 5% CO_2. Experiments were performed in triplicates (n=4).

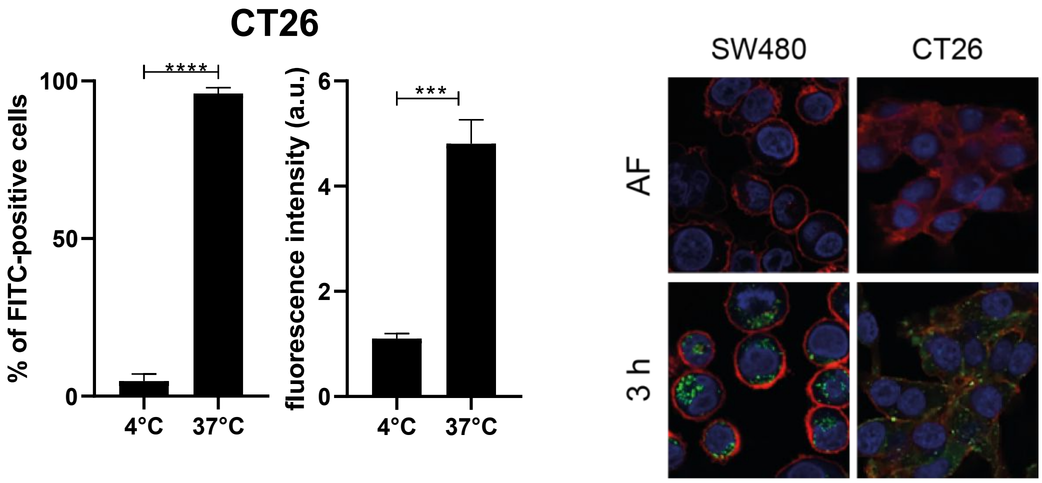

Figure S20. Cellular FITC-labeled HSA uptake in murine CT26 tumor cells. Cells were treated with 5 µM FITC-labeled HSA up to 3 h. Fluorescence was measured either by A) flow cytometry or B) fluorescence microscopy (green: BSA, blue: nuclei, red: membrane). Values given in A) are means ± SD of measured fluorescence intensity normalized to the auto fluorescence (AF) control.

VII. Animal Studies

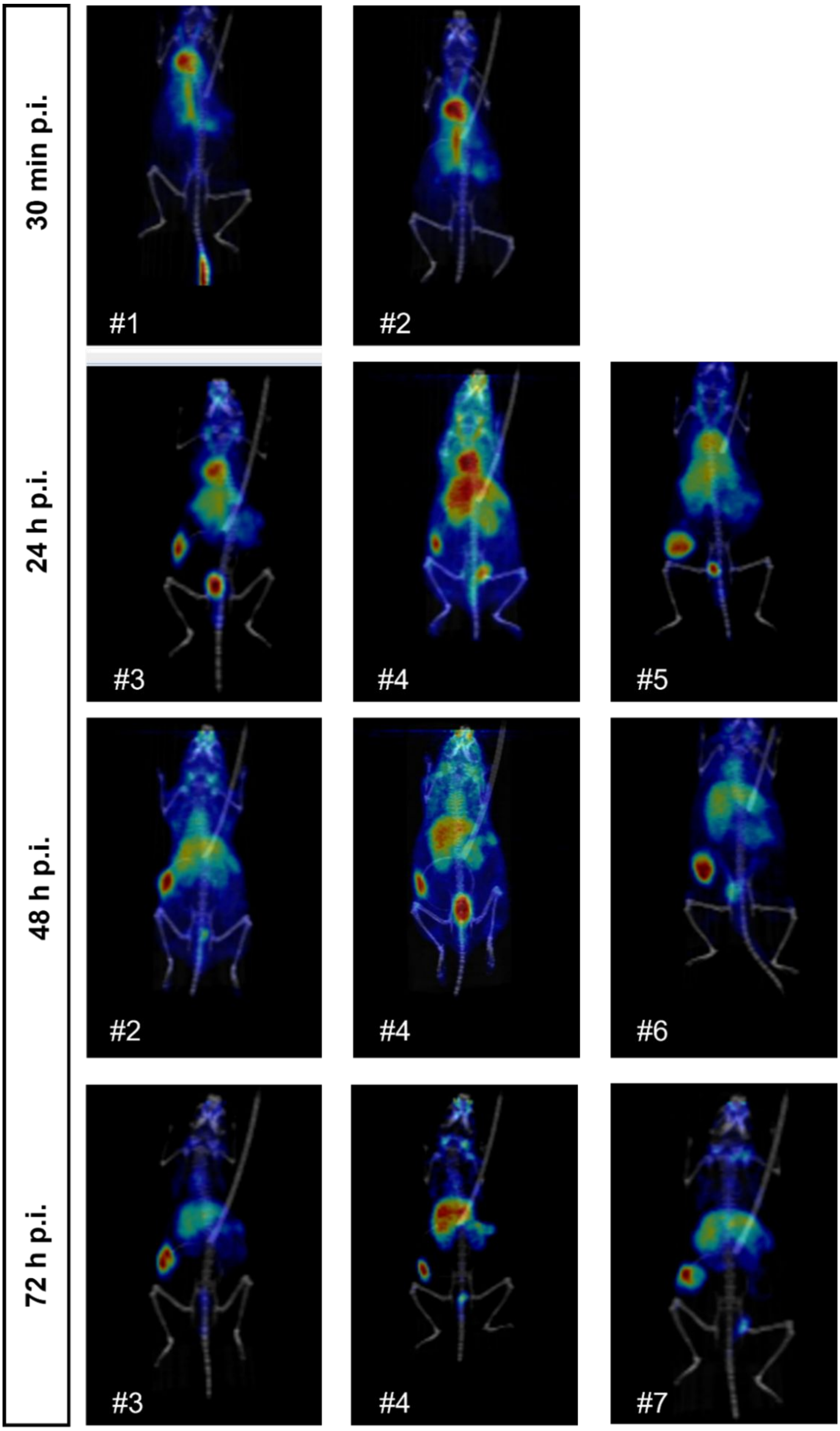

Figure S21: PET MIP fused with CT for different time points. PET data is decay corrected for PET acquisition start. Injected activities are given for the time of application: 3.39 MBq (mouse #1), 7.63 MBq (mouse #2), 10.87 MBq (mouse #3), 7.41 MBq (mouse #4), 7.41 MBq (mouse #5), 7.08 MBq (mouse #6), 12.37 MBq (mouse #7).

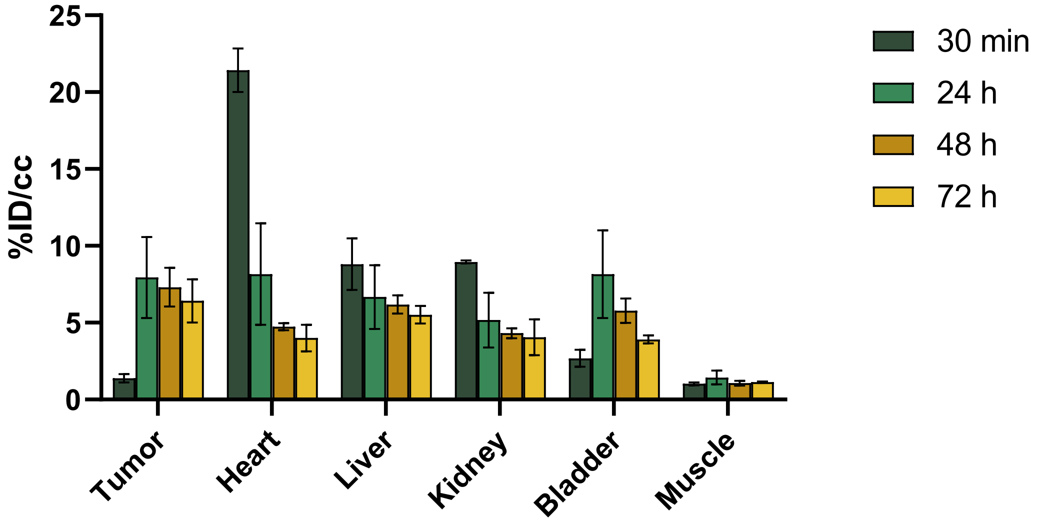

Figure S22: PET image quantification for selected organs (ID%/cc) at different time points (30 min (n=2), 24 h, 48 h, 72 h (n=3 each)). Imaging data was corrected for the start of PET acquisition.

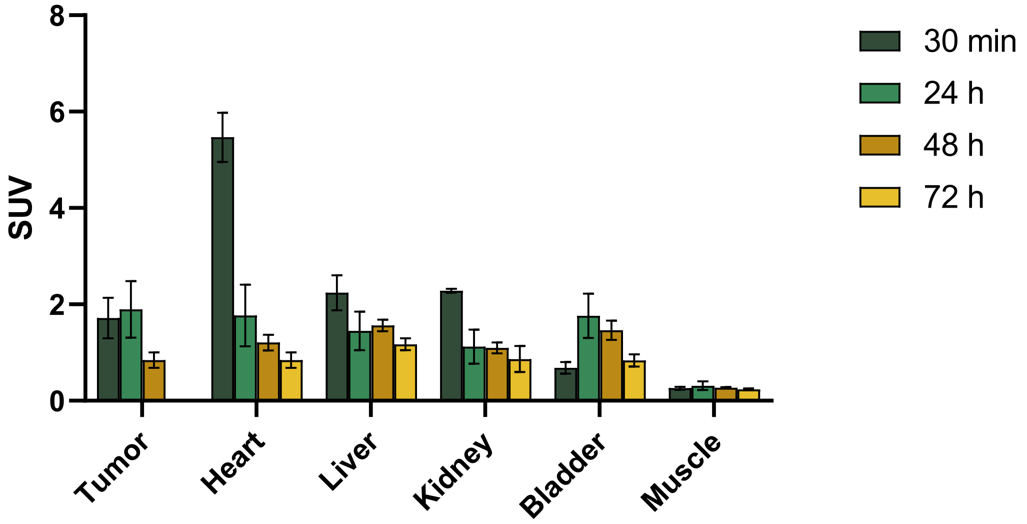

Figure S23: Standardized uptake values represent %ID/cc normalized to body weight at different time points (30 min (n=2), 24 h, 48 h, 72 h (n=3 each)). Imaging data was corrected for the start of PET acquisition.

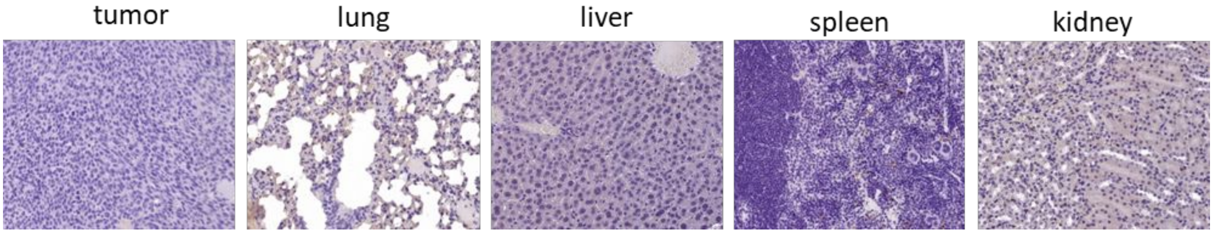

Figure S24: Proof of human specificity of the anti-HSA HRP-conjugated primary antibody (A80-129P, Biomol) on tissues of non-HSA-treated CT26-bearing Balb/C mice. Tissue sections were immune histologically stained with the anti-HSA antibody and nuclei counter-stained with hematoxylin.

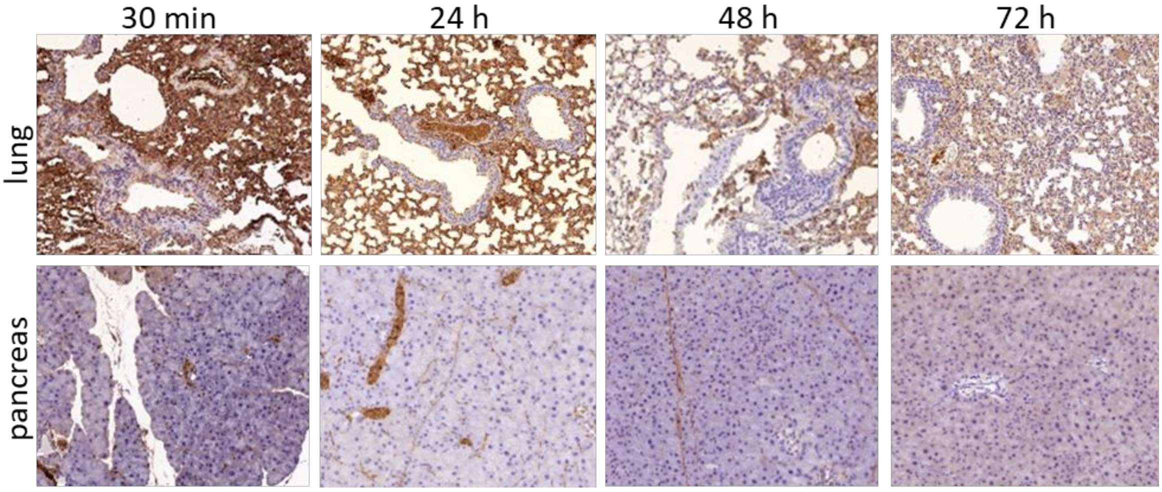

Figure S25: Representative histological staining against HSA of lung and pancreas of Balb/c mice bearing CT26 allografts. Tumor-bearing animals were treated with HSA 1 (1 g/kg) i.v. After 30 min, 24 h, 48 h and 72 h (n=3 per time point) tumors and different organs were harvested and further processed for HSA specific immunohistological staining.

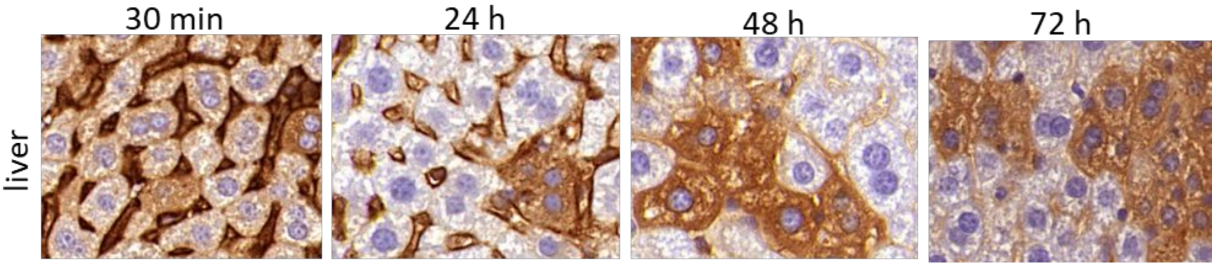

Figure S26: Representative histological staining of intracellular HSA in liver tissue of bearing CT26 Balb/c mice over time. Tumor-bearing animals were treated with HSA 1 (1 g/kg) i.v. After 30 min, 24 h, 48 h and 72 h (n=3 per time point) livers were harvested and further processed for HSA-specific immune-histological staining.

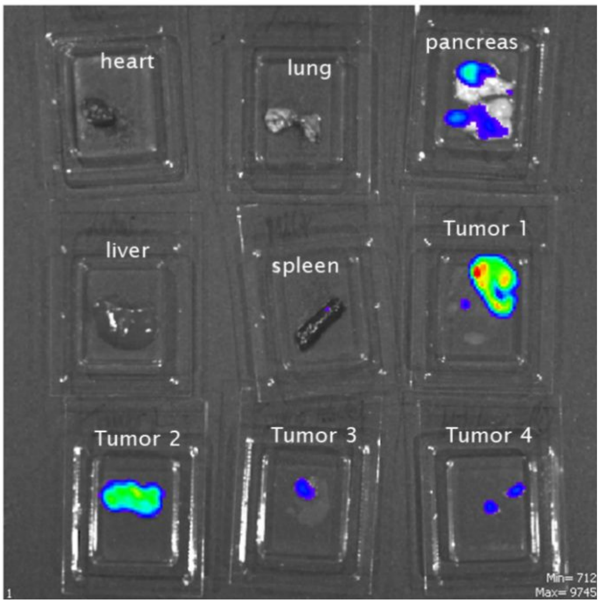

Figure S27: Post-mortem and *ex vivo* organ bioluminescence imaging of orthotopic tumor model (n = 1). Animals were sacrificed 15 min after injection of D-luciferin (150 mg kg^{-1}) and images were acquired using an IVIS imaging system.

Table S1: Radioactivity detected in joints (average of left and right) of the three animals imaged 72 hours p.i.

Animal ID	VOI (joints)	%ID/cc (corrected for PET start)	
		average	SD
Mouse 1	knee	1.797	0.599
	elbow	1.175	0.832
Mouse 2	knee	1.329	0.435
	elbow	0.921	0.397
Mouse 3	knee	1.400	0.627
	elbow	1.898	0.546

Table S2: Pooled biodistribution data of subcutaneous CT26 tumor model obtained at different timepoints *via* gamma-counter measurement and calculation of %ID g^{-1} tissue. Decay correction was performed for the time of injection of [^{89}Zr]Zr-DFO*malHSA.

pooled	30 min		24 h		48 h		72 h	
	%ID g^{-1}	n	%ID g^{-1}	n	%ID g^{-1}	n	%ID g^{-1}	n
Blood	30.1 ± 4.6	4	8.2 ± 2.3	4	3.9 ± 0.3	5	2.4 ± 0.5	6
Tumor	1.6 ± 0.6	4	9.1 ± 1.8	4	10.6 ± 3.0	4	7.6 ± 1.6	6
Heart	7.6 ± 1.2	4	3.8 ± 0.3	4	3.5 ± 0.4	5	3.0 ± 0.6	6
Lung	6.9 ± 1.0	4	3.5 ± 0.3	3	3.1 ± 0.3	5	2.6 ± 1.0	6
Liver	4.9 ± 0.2	4	5.2 ± 0.5	4	6.2 ± 0.5	5	6.1 ± 0.7	6
Spleen	4.4 ± 0.6	4	4.0 ± 0.3	4	5.4 ± 2.3	5	6.6 ± 2.5	6
Kidney	9.9 ± 1.5	4	5.8 ± 0.6	4	5.3 ± 0.9	5	5.3 ± 0.8	6
Stomach	1.0 ± 0.1	4	1.3 ± 0.1	4	1.1 ± 0.2	5	0.9 ± 0.2	6
Pancreas	1.5 ± 0.5	4	1.5 ± 0.2	4	1.4 ± 0.2	5	1.4 ± 0.2	6
Intestine	4.5 ± 2.1	4	2.0 ± 0.5	4	1.6 ± 0.4	5	1.3 ± 0.3	6
Colon	0.9 ± 0.2	4	1.4 ± 0.2	4	1.3 ± 0.4	5	1.3 ± 0.2	6
Brain	0.6 ± 0.2	4	0.2 ± 0.0	4	0.1 ± 0.0	5	0.1 ± 0.0	6
Eyes	0.8 ± 0.5	4	0.8 ± 0.1	4	0.7 ± 0.1	5	0.5 ± 0.1	6
Muscle	0.3 ± 0.1	4	0.8 ± 0.2	4	0.6 ± 0.2	5	0.6 ± 0.1	6
Bone	1.6 ± 0.5	4	1.4 ± 0.2	4	1.4 ± 0.3	5	1.7 ± 0.4	6

Table S3: Biodistribution data of subcutaneous CT26 tumor model obtained at different timepoints *via* gamma-counter measurement and calculation of %ID g^{-1} tissue. Decay correction was performed for the time of low-dose injection of [^{89}Zr]Zr-DFO*malHSA (0.27 - 1.6 MBq and 2.2 - 10.5 µg).

low dose	**30 min**		**24 h**		**48 h**		**72 h**	
	%ID g^{-1}	n	%ID g^{-1}	n	%ID g^{-1}	n	%ID g^{-1}	n
Blood	31.3 ± 4.8	3	8.5 ± 2.8	3	3.8 ± 0.4	3	2.2 ± 0.3	3
Tumor	1.8 ± 0.6	3	9.8 ± 1.4	3	10.3 ± 2.8	2	7.3 ± 0.8	3
Heart	7.6 ± 1.4	3	3.8 ± 0.4	3	3.6 ± 0.4	3	2.8 ± 0.2	3
Lung	6.9 ± 1.2	3	3.5 ± 0.3	3	2.9 ± 0.1	3	2.1 ± 0.3	3
Liver	4.9 ± 0.3	3	5.3 ± 0.5	3	6.2 ± 0.6	3	5.5 ± 0.2	3
Spleen	4.6 ± 0.5	3	4.0 ± 0.4	3	4.6 ± 2.3	3	4.7 ± 0.6	3
Kidney	10.1 ± 1.8	3	6.0 ± 0.4	3	5.0 ± 1.0	3	4.6 ± 0.4	3
Stomach	0.9 ± 0.1	3	1.3 ± 0.0	3	1.1 ± 0.1	3	0.7 ± 0.0	3
Pancreas	1.5 ± 0.7	3	1.5 ± 0.2	3	1.4 ± 0.3	3	1.4 ± 0.2	3
Intestine	5.3 ± 1.6	3	2.2 ± 0.4	3	1.4 ± 0.3	3	1.1 ± 0.2	3
Colon	0.9 ± 0.2	3	1.4 ± 0.2	3	1.1 ± 0.4	3	1.2 ± 0.1	3
Brain	0.6 ± 0.2	3	0.2 ± 0.0	3	0.1 ± 0.0	3	0.1 ± 0.0	3
Eyes	1.0 ± 0.3	3	0.8 ± 0.1	3	0.6 ± 0.0	3	0.5 ± 0.1	3
Muscle	0.3 ± 0.1	3	0.9 ± 0.2	3	0.6 ± 0.1	3	0.6 ± 0.1	3
Bone	1.6 ± 0.6	3	1.4 ± 0.2	3	1.3 ± 0.1	3	1.5 ± 0.2	3

Table S4: Biodistribution data of subcutaneous CT26 tumor model obtained at different timepoints *via* gamma-counter measurement and calculation of %ID g^{-1} tissue. Decay correction was performed for the time of high-dose injection of [^{89}Zr]Zr-DFO*malHSA (3.1 - 12.4 MBq and 26 - 78 µg).

high dose	**30 min**		**24 h**		**48 h**		**72 h**	
	%ID g^{-1}	n	%ID g^{-1}	n	%ID g^{-1}	n	%ID g^{-1}	n
Blood	26.6	1	7.5	1	4.0 ± 0.2	2	2.5 ± 0.7	3
Tumor	1.1	1	6.9	1	10.8 ± 4.4	2	7.9 ± 2.4	3
Heart	7.9	1	3.8	1	3.2 ± 0.1	2	3.2 ± 0.9	3
Lung	6.8	1	-	-	3.4 ± 0.1	2	3.2 ± 1.1	3
Liver	4.8	1	5.0	1	6.2 ± 0.4	2	6.6 ± 0.5	3
Spleen	3.6	1	4.1	1	6.6 ± 2.4	2	8.5 ± 2.1	3
Kidney	9.4	1	5.1	1	5.9 ± 0.2	2	5.9 ± 0.4	3
Stomach	1.0	1	1.1	1	1.2 ± 0.3	2	1.0 ± 0.1	3
Pancreas	1.4	1	1.3	1	1.4 ± 0.3	2	1.3 ± 0.2	3
Intestine	2.1	1	1.4	1	1.9 ± 0.5	2	1.5 ± 0.4	3
Colon	0.8	1	1.5	1	1.5 ± 0.5	2	1.3 ± 0.3	3
Brain	0.6	1	0.2	1	0.1 ± 0.0	2	0.1 ± 0.0	3
Eyes	0.3	1	0.8	1	0.7 ± 0.1	2	0.6 ± 0.1	3
Muscle	0.3	1	0.7	1	0.7 ± 0.2	2	0.7 ± 0.1	3
Bone	1.3	1	1.2	1	1.7 ± 0.3	2	2.0 ± 0.2	3

Table S5: Biodistribution data of orthotopic tumor model obtained after 48 h *via* gamma-counter measurement and calculation of %ID g^{-1} tissue (n = 1). Decay correction was performed for the time of injection of [^{89}Zr]Zr-DFO*malHSA.

	Blood	Tumor 1	Tumor 2	Tumor 3	Tumor 4	Heart	Lung	Liver	Spleen	Kidney	Stomach	Pancreas	Intestine	Colon	Brain	Eyes	Muscle	Bone
%ID g^{-1}	3.3	8.1	6.4	6.9	7.0	3.1	3.1	5.9	7.1	4.7	1.1	5.0	0.4	1.4	0.1	0.2	0.7	1.8
